# Identification of potential diagnostic targets and therapeutic strategies for anoikis-related biomarkers in lung squamous cell carcinoma using machine learning and computational virtual screening

**DOI:** 10.3389/fphar.2025.1500968

**Published:** 2025-02-14

**Authors:** Xin Zhang, Jing Zou, Jinghua Ning, Yanhong Zhao, Run Qu, Yuzhe Zhang

**Affiliations:** ^1^ College of Basic Medical sciences, Dali University, Dali, China; ^2^ Department of Respiratory Medicine, First Affiliated Hospital of Dali University, Dali, China; ^3^ Key Laboratory of Insect Biomedicine, Dali, Yunnan, China; ^4^ Key Laboratory of Anti-Pathogen Medicinal Plants Screening, Dali, Yunnan, China

**Keywords:** lung squamous cell carcinoma, anoikis, CSNK2A1, virtual screening, machine learning

## Abstract

**Objective:**

Lung squamous cell carcinoma (LUSC) is a common subtype of non-small cell lung cancer (NSCLC) characterized by high invasiveness, high metastatic potential, and drug resistance, resulting in poor patient prognosis. Anoikis, a specific form of apoptosis triggered by cell detachment from the extracellular matrix (ECM), plays a crucial role in tumor metastasis. Resistance to anoikis is a key mechanism by which cancer cells acquire metastatic potential. Although several studies have identified biomarkers related to LUSC, the role of anoikis-related genes (ARGs) remains largely unexplored.

**Methods:**

Anoikis-related genes were obtained from the Harmonizome and GeneCards databases, and 222 differentially expressed genes (DEGs) in LUSC were identified via differential expression analysis. Univariate Cox regression analysis identified 74 ARGs significantly associated with survival, and a prognostic model comprising 8 ARGs was developed using LASSO and multivariate Cox regression analyses. The model was internally validated using receiver operating characteristic (ROC) curves and Kaplan-Meier (K-M) survival curves. Differences in immune cell infiltration and gene expression between high- and low-risk groups were analyzed. Virtual drug screening and molecular dynamics simulations were performed to evaluate the therapeutic potential of CSNK2A1, a key gene in the model. Finally, *in vitro* experiments were conducted to validate the therapeutic effects of the identified drug on LUSC.

**Results:**

The 8-gene prognostic model demonstrated excellent predictive performance and stability. Significant differences in immune cell infiltration and immune microenvironment characteristics were observed between the high- and low-risk groups, suggesting the critical role of ARGs in shaping the immune landscape of LUSC. Virtual drug screening identified Dihydroergotamine as having the highest binding affinity for CSNK2A1. Molecular dynamics simulations confirmed that the CSNK2A1-Dihydroergotamine complex exhibited strong binding stability. Further *in vitro* experiments demonstrated that Dihydroergotamine significantly inhibited LUSC cell viability, migration, and invasion, and downregulated CSNK2A1 expression.

**Conclusion:**

This study is the first to construct an anoikis-related prognostic model for LUSC, highlighting its role in the tumor immune microenvironment and providing insights into personalized therapy. Dihydroergotamine exhibited significant anti-LUSC activity and holds promise as a potential therapeutic agent. CSNK2A1 emerged as a robust candidate for early diagnosis and a therapeutic target in LUSC.

## 1 Introduction

Lung cancer is one of the most prevalent malignancies worldwide. According to the 2022 Global Cancer Statistics, it remains a leading cause of cancer-related deaths, with a 5-year survival rate of less than 15% (Xia et al., 2022). Lung squamous cell carcinoma (LUSC) a subtype of non-small cell lung cancer (NSCLC). Accounts for approximately 30% of NSCLC cases and is characterized by high recurrence and metastasis rates (Qian et al., 2016). Currently, the standard treatment for LUSC in involves the use of immune checkpoint inhibitors in combination with carboplatin and paclitaxel (Fan et al., 2019). While these methods can extend progression-free survival, overall outcomes remain suboptimal (Pan et al., 2021; Gao et al., 2020). Consequently, there is an urgent need to further investigate the pathophysiology of LUSC and identify reliable biomarkers for improved diagnosis and treatment. Although numerous studies have explored genes related to LUSC prognosis, the role of anoikis-related genes (ARGs) in LUSC has not been adequately investigated. Therefore, we conducted a preliminary investigation into the potential use of ARGs as novel biomarkers for LUSC.

Anoikis is a specialized form of apoptosis that occurs when cells lose proper attachment to the extracellular matrix (ECM) or neighboring cells (Taddei et al., 2012). Under normal physiological conditions, anoikis is triggered when cells detach from the ECM. leading to cell apoptosis. However, cancer cells that develop resistance to anoikis can survive despite detachment, Thereby, evading isolation-induced apoptosis. This anoikis resistance enhances the ability of cancer cells to metastasize and is considered a critical step in tumor progression (Zhang et al., 2023). Studies have demonstrated that Anoikis resistance plays a key role in the metastasis of lung adenocarcinoma (LUAD) (Chunhacha et al., 2012; Prateep et al., 2018). However, its role in LUSC remains unclear. This study was designed to explore the significance of anoikis resistance in LUSC.

**FIGURE 1:**
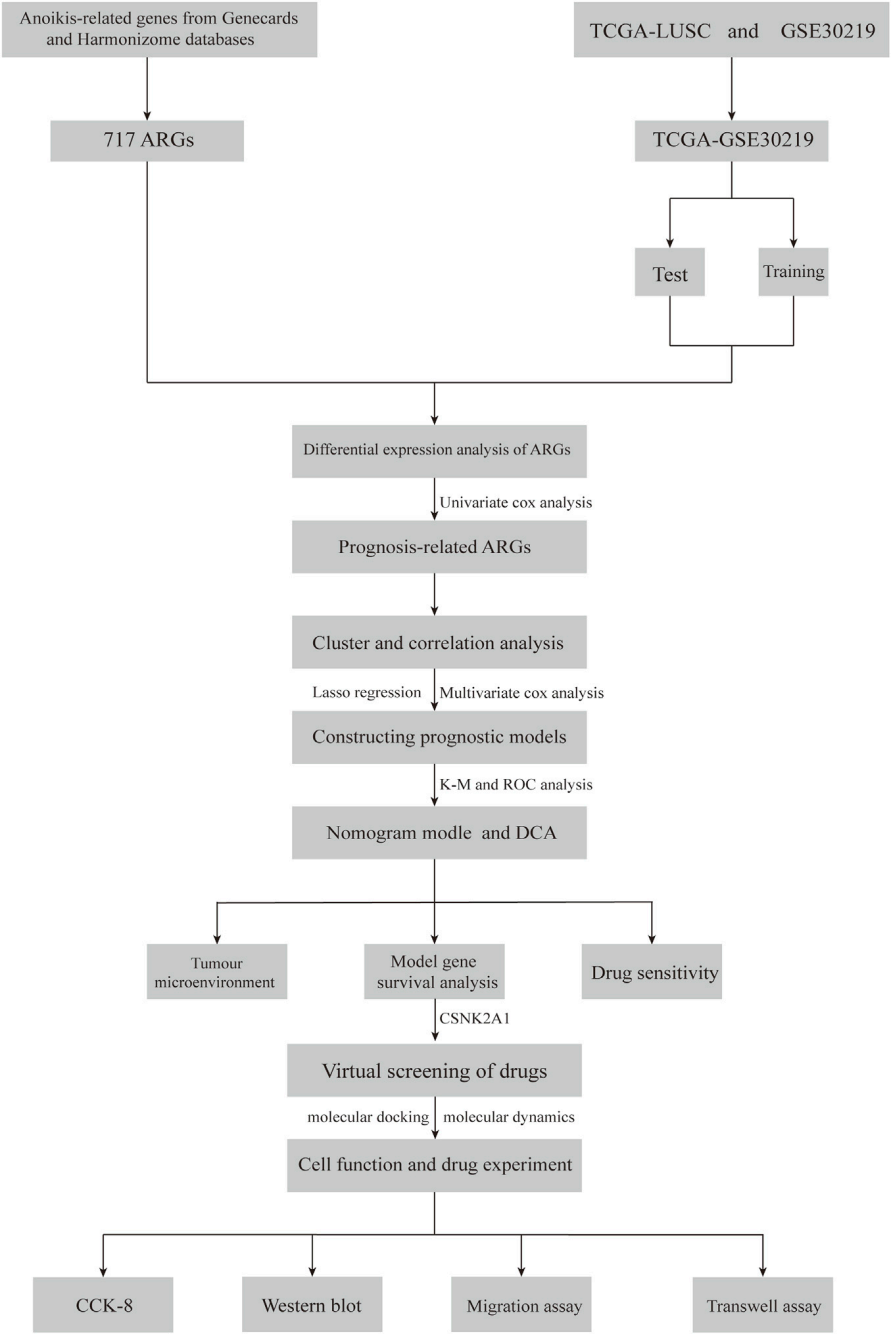
Flowchart of this study.

In this study, we first analyzed the distribution of ARGs in LUSC and developed a risk model based on ARGs to predict the prognosis of LUSC patients and the sensitivity of immunotherapy. Additionally, we validated the reliability of genes-drugs interactions through virtual screening and molecular dynamics modeling. The flow of the study is shown in Figure 1.

## 2 Methods

### 2.1 Data sources

Transcriptomic and clinical data for LUSC were obtained from the Gene Expression Omnibus (GEO) (https://www.ncbi.nlm.nih.gov/geo/) and The Cancer Genome Atlas (https://www.cancer.gov/ccg/research/genome-sequencing/tcga) databases. The GSE30219 dataset contains 82 LUSC samples and 14 normal samples, while the TCGA dataset includes 502 LUSC samples and 51 normal samples. Additionally, we retrieved anoikis-related genes (ARGs) from the Harmonizome portals (Rouillard et al., 2016) and GeneCards (https://www.genecards.org/) portals (Rebhan et al., 1997).

### 2.2 Differential gene identification and prognostic analysis

Differential expressed genes (DEGs) were identified by comparing ARGs expression in normal and tumor tissues within the TCGA-LUSC cohort. Prognostic relevance to LUSC was then assessed using univariate Cox regression analysis. To create a more robust dataset, The TCGA-LUSC cohort was combined with the GSE30219 dataset, followed by batch effect correction, resulting in the integrated to obtain the new TCGA-GSE30219 cohort.

### 2.3 Cluster analysis

Based on the prognostically relevant ARGs, each LUSC patient in the TCGA-GSE30219 cohort was assigned an anoikis score using the Gene Set Variation Analysis (GSVA) algorithm. Patients were subsequently divided into high-and low-score groups by determining cut-off values for the scores using the ‘survminer’ package. Uniform Manifold Approximation and Projection (UMAP) and t-distributed stochastic neighbor embedding (t-SNE) were employed to ensure clustering consistency. The final clustering results were visualized with the ‘ggplot2′ package.

### 2.4 Functional enrichment analysis of ARGs

To investigate the potential functional mechanism of ARGs in LUSC, we downloaded the “c2. cp.Kegg.symbols.gmt” file from the Molecular Signatures Database (MSigDB) (Liberzon et al., 2015). The biological pathways and functions related to ARGs were then analyzed using Gene Set Variation Analysis (GSVA) through with the “GSVA” package.

### 2.5 Construction of an ARGs-Based prognostic model for LUSC

The ARGs associated with LUSC prognosis from the integrated TCGA-GSE30219 cohort were randomly split into a training set and a validation set, each comprising 50% of the data. Survival-related genes were identified using the Least Absolute Shrinkage (LASSO) regression algorithm, with the regularization parameter λ determined via 10-fold cross-validation (Ding et al., 2023). These gene expression levels were then used to assess the survival prognosis of LUSC patients in the training set. The optimal model was selected through multivariate Cox regression analysis, and coefficients for the model genes were calculated. The risk score was derived using the formula: risk score = ∑(Exp (mRNA) × coef (mRNA)), where Exp represents gene expression and coef denotes the gene’s coefficient. The model’s predictive accuracy was validated using time-dependent ROC curves and Kaplan-Meier (KM) survival curves.

### 2.6 Relationship between risk score and immune infiltration

The relative proportion of immune cell infiltration was estimated using single-sample gene set enrichment analysis (ssGSEA) and “CIBERSORT” algorithm (Newman et al., 2015). Immune cell proportions in the high-and low-risk groups were analysed using the ‘CIBERSORT’ package. Where the sum of the proportions of all immune cell types in each sample was normalized to 1. The relationship between the risk score and immune cell infiltration was subsequently evaluated through Spearman’s rank correlation analysis.

### 2.7 Stratified analysis based on clinicopathological features

To validate the risk model as an independent prognostic indicator, we first assessed the correlation between overall survival (OS) and clinicopathological characteristics-such as age, gender, pathological stage, and risk score-using univariate Cox regression analysis. Independent prognostic factors for LUSC were then identified through multivariate Cox regression analysis. A nomogram was constructed based on the risk score and clinicopathological features. The accuracy of the nomogram was evaluated using calibration plots and time-dependent concordance index (Time-C index), Additionally, the clinical benefit of the nomogram was assessed through decision curve analysis (DCA) (Kerr et al., 2016).

## 3 LUSC model gene analysis and drug sensitivity

### 3.1 LUSC drug sensitivity projections

Using data from the Cancer Treatment Response Portal (CTRP), the ‘oncoPredict’ package was employed to predict the 50% inhibitory concentration (IC50) of LUSC samples for various antineoplastic drugs. Spearman’s correlation analysis was then performed between the IC50 value and the risk score to identify drug sensitivity resistance in LUSC (P < 0.05) (Maeser et al., 2021).

### 3.2 Virtual screening and molecular dynamics simulation

To validate the potential of model genes as therapeutic targets for LUSC, further analyses were conducted on the genes associated with LUSC survival. Protein structure files were downloaded from the Protein Data Bank (PDB) database (https://www.rcsb.org/), and water molecules and ligands were removed using PyMOL 2.3.0. The docking regions were identified using the getbox plugin, and the protein files were hydrogenated in AutodockTools, and saved in pdbqt format for virtual screening. Small molecule drugs approved by the US Food and Drug Administration (FDA) were selected from the ZINC15 database (https://zinc15.docking.org/). These drug files were processed using Open Babel and screened virtually using Autodock vina, set to semi-flexible docking docking with exhaustiveness = 25 (Trott and Olson, 2010; Eberhardt et al., 2021). Drugs with the strongest affinity to the target gene were selected. Since semi-flexible docking does not consider factors like protein flexibility, temperature, pressure, or solvent effect, 100ns molecular dynamics simulations of the protein-ligand complexes were conducted using Gromacs2022. Amber14sb was used as the protein force field, Gaff2 as the ligand force field, and the SPC/E water model was used to solvents the system with a periodic boundary of 1.2 nm. The particle mesh Ewald (PME) method was used for electrostatic interactions, and the Mont Carlo method for ion placement, neutralizing the system’s charge with appropriate amounts of sodium and chloride ions. Before running the simulation, the system underwent three energy minimization and equilibration steps: (1) Energy minimisation was performed using the steppest descent algorithm for 50,000 steps. (2) A 50,000-step pre-equilibration with a 2fs timestep was performed, maintaining constant particle number, volume, pressure, temperature (310 K). After energy minimization and equilibration, unconstrained molecular dynamics simulations were conducted for 100 ns with a 2 fs timestep. The stability of the complexes was evaluated by analysis the root-mean-square deviation (RMSD) of the molecular-dynamics trajectories. A smaller RMSD indicates less structural deviation within the complexes (Mukherjee et al., 2010); Root-mean-square fluctuation (RMSF) was used to assess fluctuations in amino acid residue, with lower RMSF values indicating greater stability. (Zhang and Zhang, 2024); The radius of gyration (Rg) was calculated to assess the compactness of the structure (Lobanov et al., 2008); In addition, we analyzed the number of hydrogen bonds between proteins and ligands, the relative free energy distributions, and structural comparisons of the complexes at 0, 25, 50, 75, and 100 ns Finally, the average binding free energy between protein and ligand was calculated using the MM/GBSA method (Liu L. et al., 2023).

### 3.3 Cell culture and drug treatment

In this study, the LUSC cell line (NCI-H2170) was obtained from Zhejiang Noble Biological Products Company (www.noblebio.cn). The cells were cultured in RPMI-1640 medium (Gibco, Life Technologies, China) supplemented with 10% fetal bovine serum (SERANA, Europe) and 1% penicillin-streptomycin (Beyotime, China). They were seeded into culture flasks (Nest, China) and incubated in a humidified atmosphere at 37°C with 5% CO₂. The dihydroergotamine standard was sourced from MCE China (https://www.medchemexpress.cn), and its working solution (100 μM) was prepared in sterile dimethyl sulfoxide (DMSO). Care was taken to ensure that the final concentration of DMSO in the medium remained below 0.1% of the total volume.

### 3.4 Cell viability assay

NCI-H2170 cells were seeded at a density of 4 × 10^3^ cells/well in 96-well plates with 100 μL of complete medium per well and incubated in a humidified atmosphere at 37°C. The drug concentrations were set at 0 μM (untreated control group), 5 μM, 10 μM, 20 μM, 40 μM, and 80 μM. The cells were incubated for 24, 48, and 72 h. A mixture of RPMI-1640 medium and CCK-8 reagent (Biosharp, China) was prepared at a ratio of 1:10. After incubation, 100 μL of the mixture was added to each well, followed by further incubation for 1 h. Finally, absorbance at 450 nm was measured using an enzyme-linked immunosorbent assay (ELISA) reader.

### 3.5 Wound migration assay

NCI-H2170 cells were seeded at a density of 1 × 10^6^ cells per well in 12-well plates and incubated overnight. Once the cells reached 95% confluence, the culture medium was removed, and a scratch was created using a 10 μL pipette tip. The cells were washed with phosphate-buffered saline (PBS) to remove debris, and images were captured at the same position under a microscope (×100 magnification). The medium was then replaced with either serum-free medium (control group) or serum-free medium containing Dihydroergotamine (40 μM) (treatment group). The cells were incubated at 37°C for 43 h in a humidified incubator. After incubation, images of the wound area were captured again at the same position. The wound area was quantified using ImageJ software, and statistical analysis was performed using Prism software.

### 3.6 Cell invasion assay

A 24-well transwell plate with 8 μm pore polycarbonate membranes (Corning, United States) was used for the cell invasion assay. Matrigel (Solarbio, Beijing, China) was diluted with RPMI-1640 medium at a ratio of 1:8, and the polycarbonate membrane was coated with 8.1 mg/mL of diluted Matrigel. The plate was incubated at 37°C for 1 h to solidify the Matrigel layer. A total of 2 × 10^4^ cells were seeded into the upper chamber in 150 μL of serum-free medium, while 500 μL of RPMI-1640 medium supplemented with 10% fetal bovine serum (FBS) was added to the lower chamber as a chemoattractant. The plate was incubated at 37°C for 48 h. After incubation, the medium in the upper chamber was removed, and the membranes were washed twice with phosphate-buffered saline (PBS). Invaded cells on the lower side of the membrane were fixed with 4% paraformaldehyde and stained with 0.1% crystal violet. Two randomly selected fields were imaged under a microscope (×100 magnification), and the number of invaded cells was counted using ImageJ software. Statistical analysis was performed with Prism software.

### 3.7 Western blot

Cell lysis buffer was prepared by mixing RIPA lysis buffer (Biyotime, China) with PMSF protease inhibitor at a ratio of 1:100. A total of 30 μg of protein was separated by 10% SDS-PAGE electrophoresis and subsequently transferred onto a PVDF membrane (Millipore, United States). The membrane was blocked with 5% skimmed milk for 1 h at room temperature and then incubated overnight at 4°C with rabbit anti-CSNK2A1 antibody (10992-1-AP, Proteintech, China) and rabbit anti-GAPDH antibody (60004-1-Ig, Proteintech, China). On the following day, the membrane was incubated with horseradish peroxidase (HRP)-conjugated secondary antibodies (goat anti-rabbit IgG/HRP and goat anti-mouse IgG/HRP, Proteintech, China) for 1 h at room temperature. Protein signals were detected using ECL detection reagents (Beyotime, China). The band intensities were analyzed using ImageJ software to quantify protein expression levels.

### 3.8 Cell invasion assay

A 24-well transwell plate with 8 μm pore polycarbonate membranes (Corning, United States) was used for the cell invasion assay. Matrigel (Solarbio, Beijing, China) was diluted with RPMI-1640 medium at a ratio of 1:8, and the polycarbonate membrane was coated with 8.1 mg/mL of diluted Matrigel. The plate was incubated at 37°C for 1 h to solidify the Matrigel layer. A total of 2 × 10^4^ cells were seeded into the upper chamber in 150 μL of serum-free medium, while 500 μL of RPMI-1640 medium supplemented with 10% fetal bovine serum (FBS) was added to the lower chamber as a chemoattractant. The plate was incubated at 37°C for 48 h. After incubation, the medium in the upper chamber was removed, and the membranes were washed twice with phosphate-buffered saline (PBS). Invaded cells on the lower side of the membrane were fixed with 4% paraformaldehyde and stained with 0.1% crystal violet. Two randomly selected fields were imaged under a microscope (×100 magnification), and the number of invaded cells was counted using ImageJ software. Statistical analysis was performed with Prism software.

### 3.9 Sensitivity analysis

The data were processed using the Perl programming language (v 5.30.0) and all analyses were performed in the R software (v4.4.0). P < 0.05 was considered statistically significant. (*, P < 0.05; **, P < 0.01; ***, P < 0.001; ****, P < 0.0001).

## 4 Results

### 4.1 Identification of prognosis-associated ARGs

**FIGURE 2:**
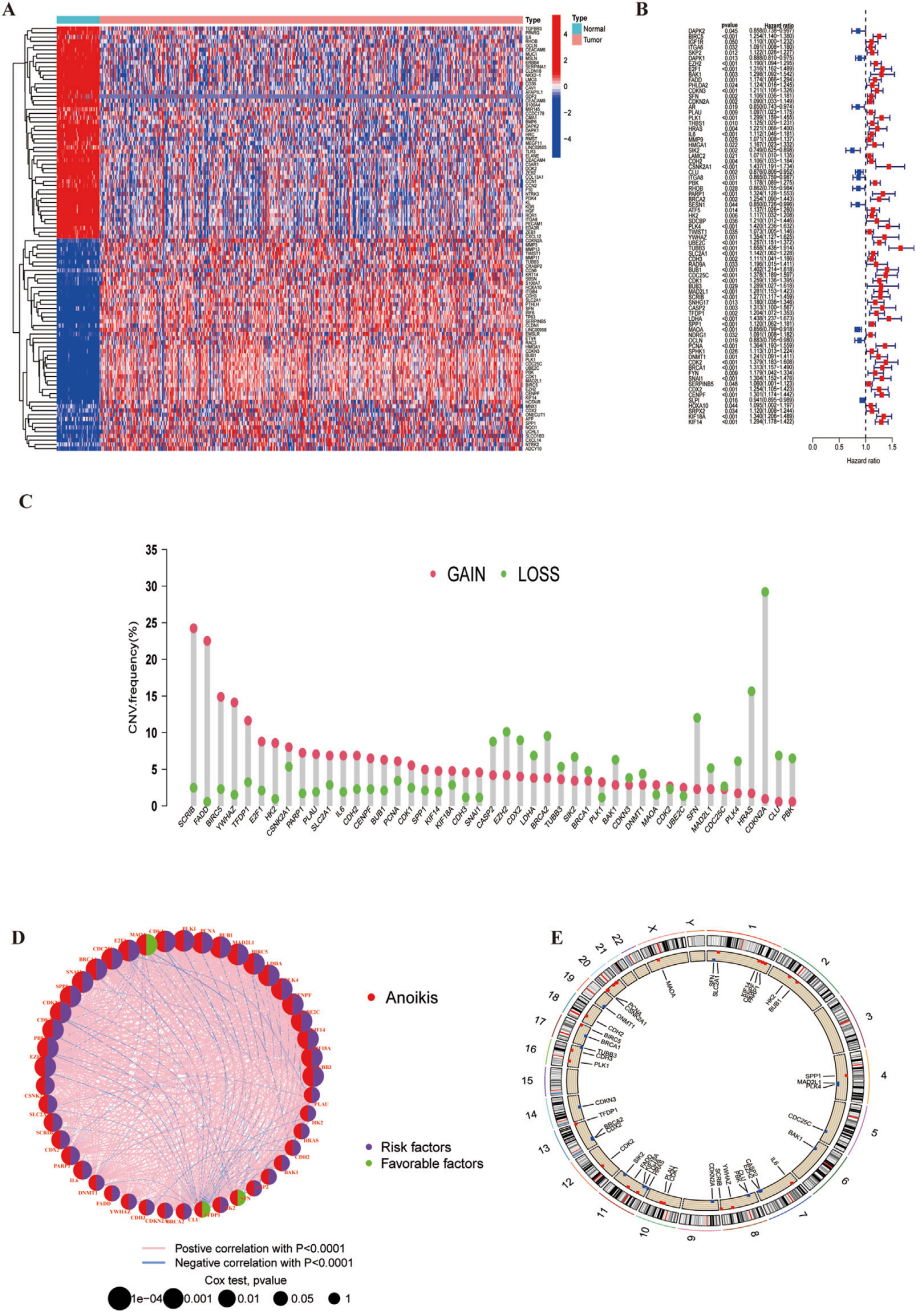
Anoikis-related differentially expressed genes and their associated regulatory factors in LUSC. **(A)** A total of 222 ARGs were identified from TCGA-LUSC cohort. **(B)** A forest plot showing the top 45 ARGs identified through univariate Cox regression analysis. **(C)** Copy number variations (CNVs) of the top 45 ARGs in the TCGA-LUSC. **(D)** Correlation network diagram among the top 45 ARGs. **(E)** Chromosomal region alterations of the ARGs.

We obtained a total of 717 ARGs from the Genecards and Harmonizome portal (Supplementary Table S1) and identified 222 DEGs differences expressed genes (DEGs) with significant expression differences in the TCGA-LUSC cohort (LogFC|>1, FDR<0.05, Supplementary Table S2)|, A heatmap was used to display the top 30 genes with the most significant expression changes (Figure 2A) Using univariate Cox regression analysis, we found that 73 of these DEGs were significant were significantly associated with LUSC survival (Supplementary Table S3, P < 0,05), The top 45 significant ARGs are shown in a forest plot (P < 0.01). Where all genes, except for MAOA,CLU, and SIK2, were associated with poor tumour prognosis (Figure 2B). The gene expression network revealed strong association between these 45 genes, suggesting their critical role in LUSC progression (Figure 2D). Additionally, we analysed the copy number variation (CNV) of these ARGs across chromosomes (Figures 2C, E; Supplementary Table S4). The results showed that CDKN2A has the most significant copy number loss on chromosome 9, while SCRIB has exhibited the most significant copy number gain on chromosome 8. Finally, we constructed protein-protein interaction (PPI) networks using the STRING (https://cn.string-db.org/) and GeneMANIA (https://genemania.org/) databases to visualize interactions (PPI) among the genes most closely related to anoikis (Supplementary Figure S1).

### 4.2 ARGs-based clustering of LUSC molecular subgroups

**FIGURE 3:**
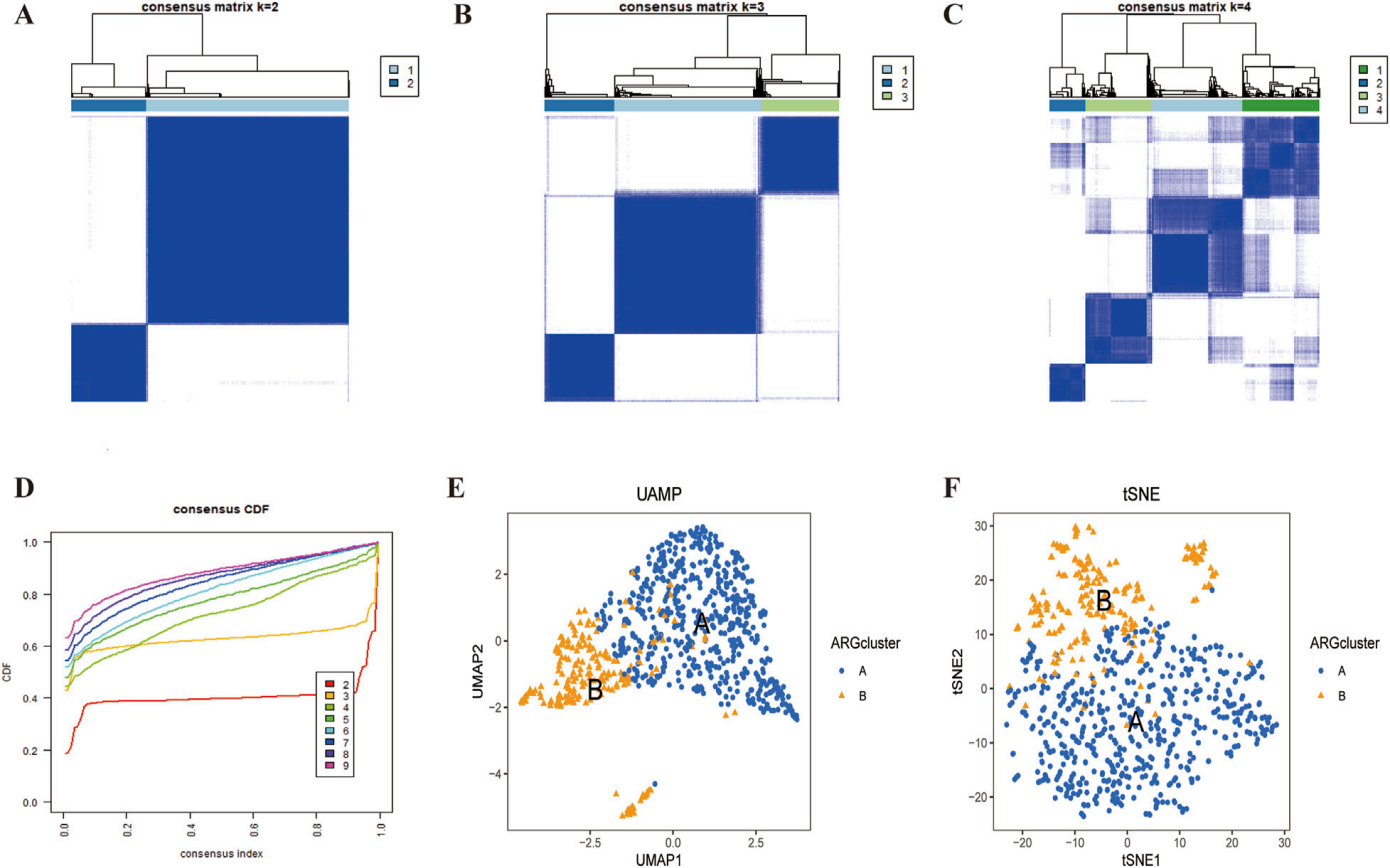
LUSC subtypes associated with ARGs. **(A–D)** A consensus matrix for k = 2 was generated through consensus clustering. **(C,D)** The two subtypes were differentiated using UMAP and t-SNE based on the expression of ARGs.

**FIGURE 4:**
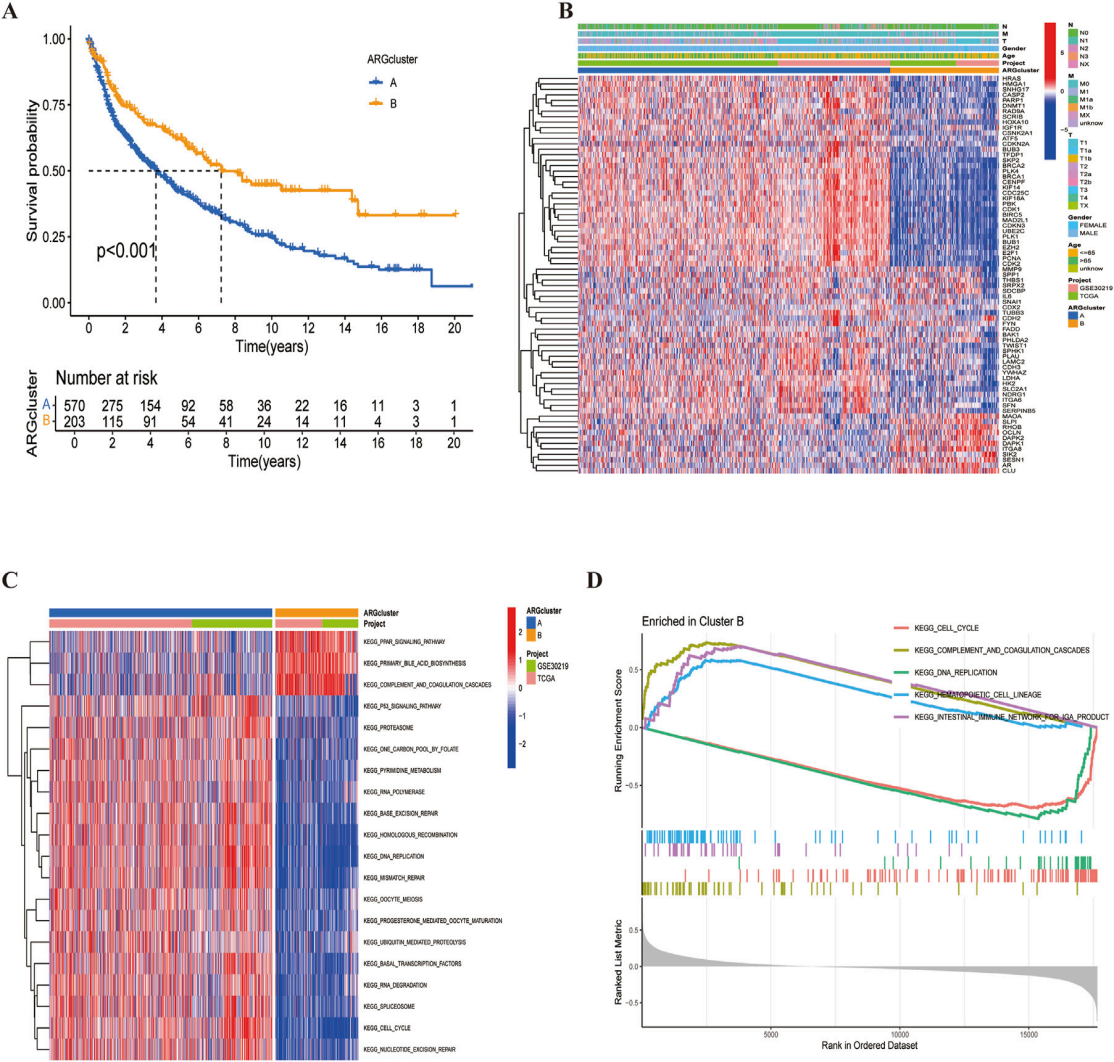
LUSC subtypes associated with ARGs. **(A)** Overall survival analysis of the two subtypes (P < 0.001). **(B)** Differential expression of ARGs between the two subtypes. **(C, D)** Different KEGG pathway enrichment levels between the two subtypes.

We used the ‘ConsensusClusterPlus’ package to consistently cluster the 74 prognostically relevant ARGs (P < 0.05). Based on the clustering results, the cohort could was clearly divided into two subtypes (Figures 3A–D) when K = 2 (Supplementary Table S5). UMAP and t-SNE analyses confirmed the high accuracy of the clustering (Figures 3E, F). Kaplan-Meier analysis showed that the Overall survival (OS) between the two subtypes was significant different (P < 0.001) (Figure 4A).

**FIGURE 5:**
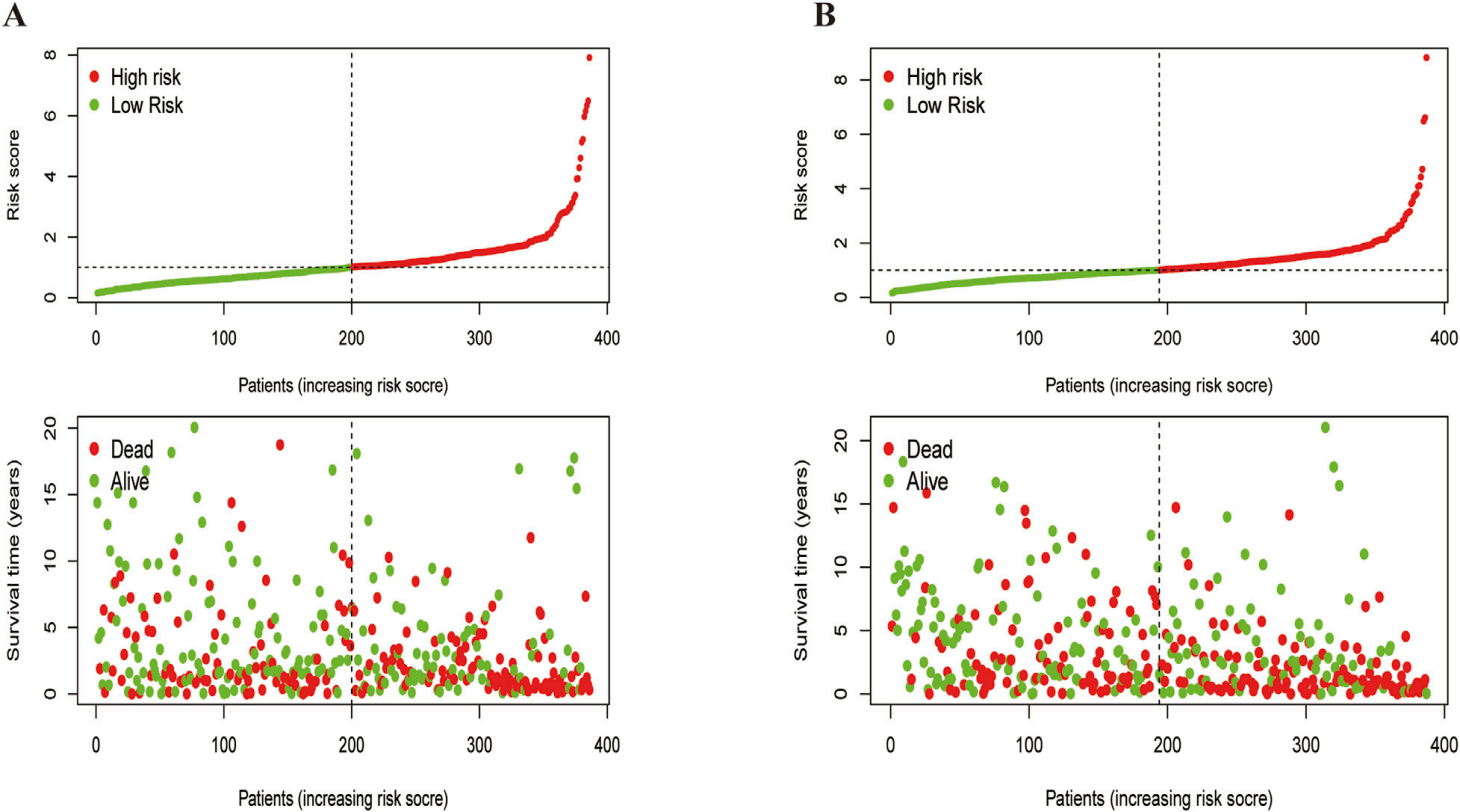
Feasibility analysis of the two subtypes models.

A heatmap revealed that CDX2 was expressed at low levels in most samples across both subtypes, potentially serving as a favorable prognostic marker (Figure 4B). Additionally, KEGG pathway enrichment analysis using the ‘GSVA’ package identified differences between cluster A and cluster B (Figures 4C, D). Cluster B exhibited a poorer prognosis, predominantly involving pathways such as complement and coagulation cascade, peroxisome proliferator-activated receptor activation, and primary bile acid biosynthesis—pathways closely related to cancer development. Finally, risk curve analysis supported the validity of the subtype classification and demonstrated that the prognosis of LUSC patients progressively worsened with an increasing score (Figure 5).

### 4.3 Analysis of differences in gene expression and immune cell infiltration between the two subtypes

**FIGURE 6:**
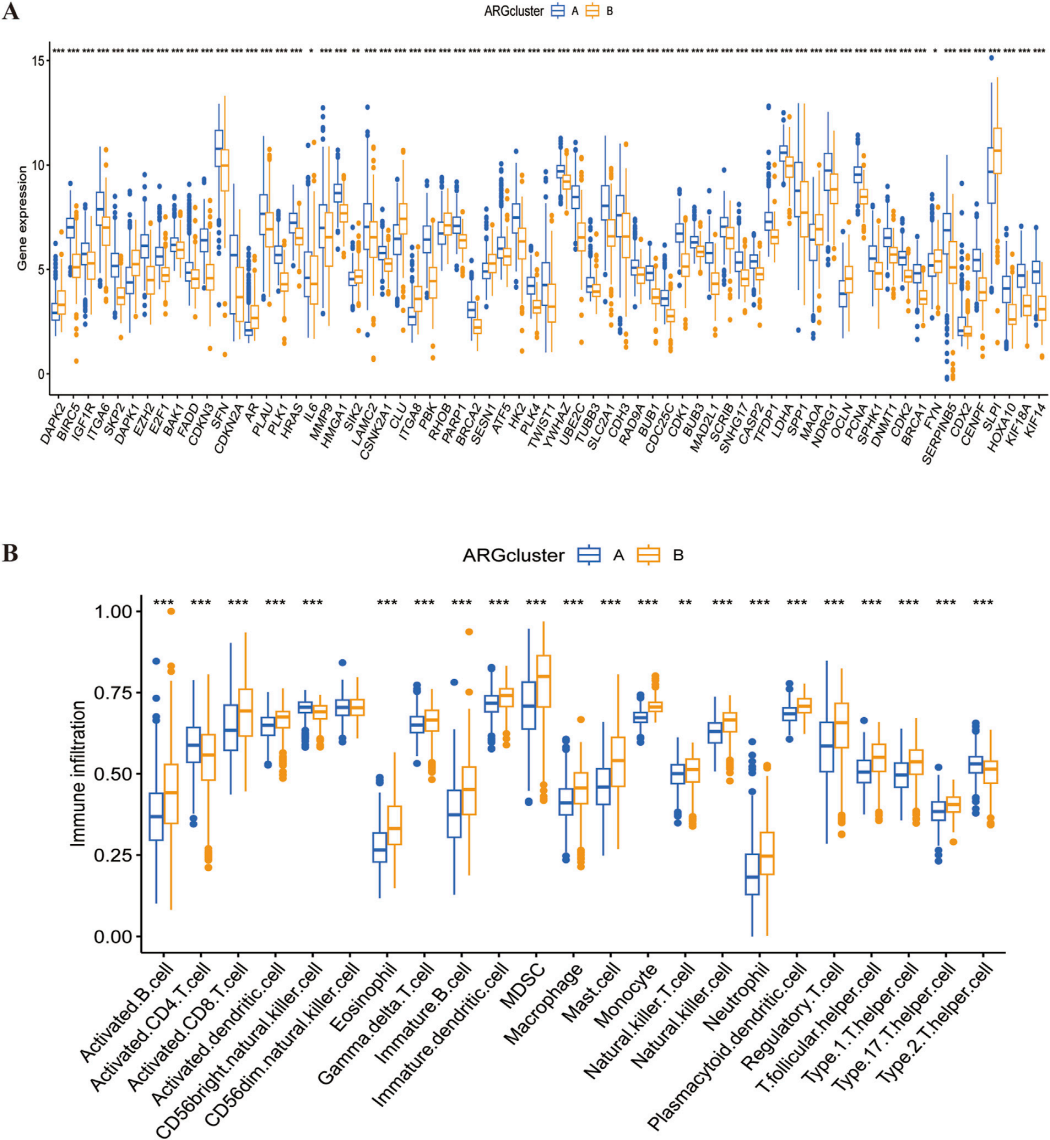
Differences in gene expression and immune infiltration between the two subtypes. **(A)** Differential expression of ARGs across the two subtypes. **(B)** Immune infiltration profiles of the two subtypes.

We demonstrated significant difference in ARGs expression (P < 0,05) between groups A and B using box-and-line plots (Figure 6A). Since group B had a poorer prognosis in earlier analysis, genes significantly downregulated in this group may have a positive effect on LUSC prognosis; Conversely, genes highly expressed in group B could indicate a worse prognosis but may serve as potential therapeutic targets. SsGSEA results showed that, except for activated CD4 T-cells, all other immune cells had a significantly higher level of infiltration in group B compared to group A (Figure 6B; Supplementary Table S6). This suggests that the poor prognosis of LUSC may be broadly linked to immune cell involvement, highlighting the potential of immunotherapy in LUSC treatment.

### 4.4 ARGs-related prognostic model construction and validation

**FIGURE 7:**
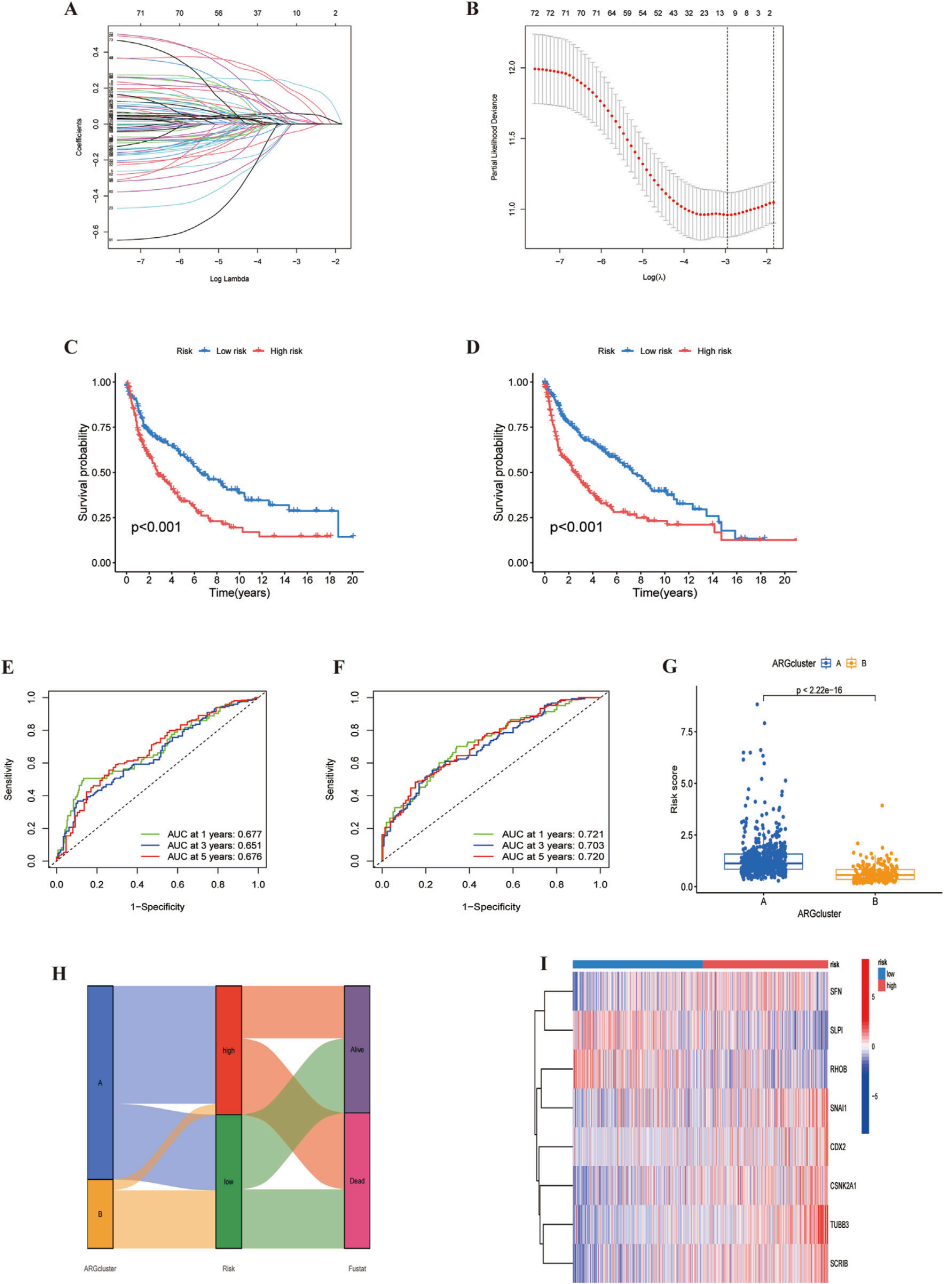
Identification of ARGs prognostic signature. **(A)** LASSO analysis with cross-validation identified 11 prognostically relevant ARGs. **(B)** Coefficients of 11 prognostically relevant ARGs. **(C, D)** Kaplan-Meier curves for two subtype risk groups. **(E, F)** Time-dependent ROC curves for 1-, 3- and 5-year OS. **(G)** Risk score distribution of ARG clusters. **(H)** Alluvial diagram showing subtypes transitions and survival status.**(I)** Heatmap of the expression patterns of the 11 ARGs.

Using univariate Cox regression analysis, we identified 11 survival-related ARGs (Figures 7A, B), followed by LASSO regression, which narrowed them down to 8 independent prognostic factors. A risk model was then constructed based on multivariate Cox regression analysis (Supplementary Table S7). The risk score was calculated as follow: risk score=(0.175×SFN expression)+(0.238×CSNK2A1 expression)+(-0.266×RHOB expression)+(0.436×TUBB3 expression +(0.165×SCRIB expression)+ (0.290×SNAI1 expression)+(0.137×CDX2 expression)+(-0.126×SLPI expression). We divided the data into training and validation groups and classified patients into high-risk and low-risk categories based on the median risk score. Kaplan-Meier (K-M) curves indicated that the high-risk group had poorer prognosis in both the training and validation groups (Figures 7C, D). Additionally, the time-dependent ROC curves at 1, 3 and 5 years for overall survival (OS) in the training and validation groups confirmed the accuracy of the model, showing significantly differences in risk scores between the two subtypes (P < 0.05) (Figures 7E–G). Sankey plots illustrated the relationship between LUSC sample clusters, risk scores, and survival status (Figure 7H). Finally, we used a heatmap to analyze the expression of eight ARGs in the high-risk and low-risk groups, demonstrating that six ARGs, except RHOB and SLPI, were highly expressed in the high-risk group (Figure 7I).

### 4.5 LUSC immunocorrelation analysis

**FIGURE 8:**
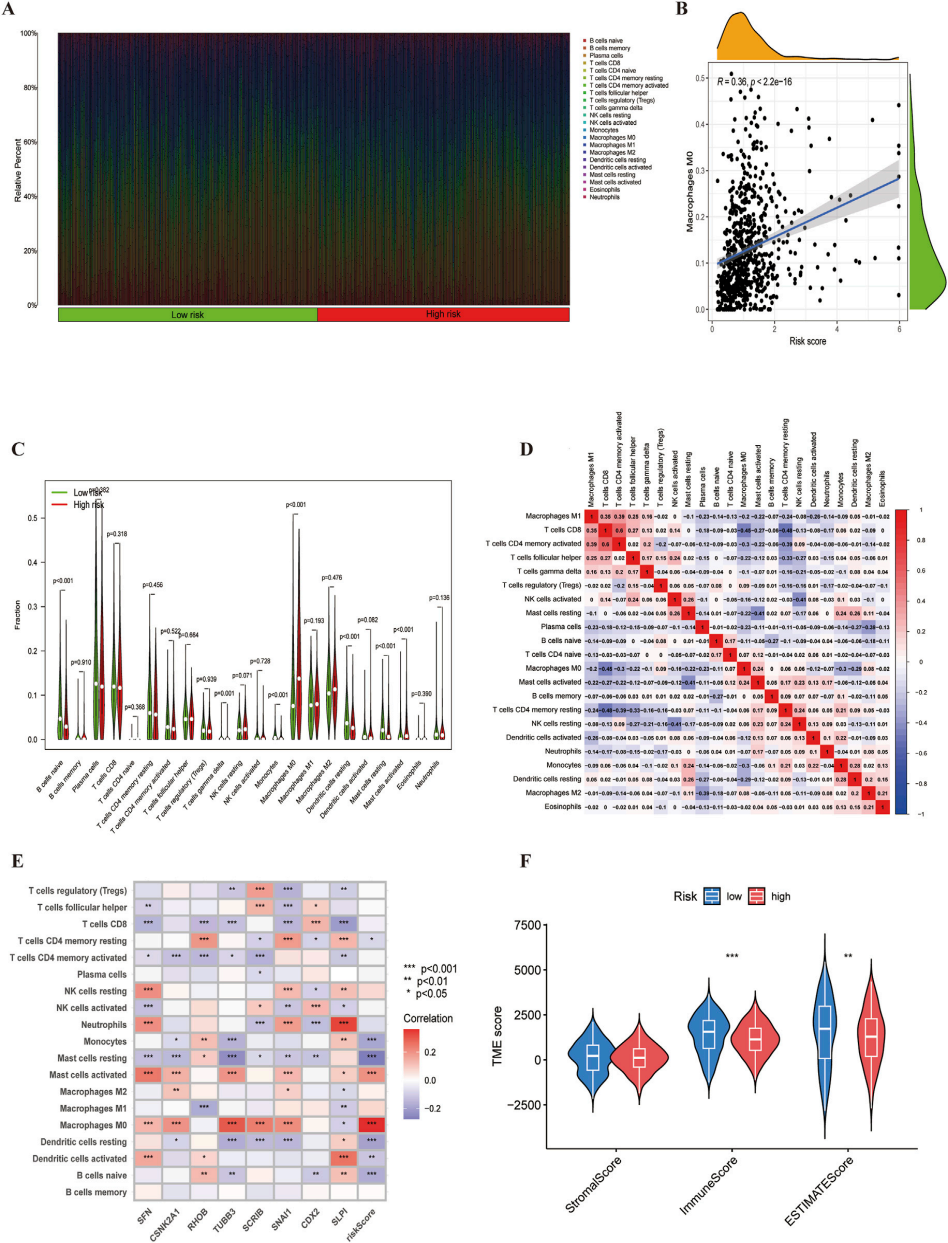
The immune microenvironment of LUSC. **(A)** Proportion of immune cell infiltration. **(B)** Correlation between risk scores with the proportion of M0 macrophage in LUSC.**(C)** Differences in immune cell populations between high-risk and low-risk groups. **(D)** Correlation analysis among immune cells.**(E)** Gene-immune cell correlation analysis. **(F)** Estimated scores for expression profiles of the two risk groups.

The tumour immune microenvironment plays a crucial role in cancer development and treatment. Using the ‘CIBERSORT’ R package, We analyzed immune cell infiltration differences between high and low risk groups. First, we visualized the distribution of immune cell counts in relation to the risk score (Figure 8A). As the score increased, the proportion of M0 macrophages also increased (Figure 8B). Monocytes and resting mast cells were more prevalent in the low-risk group, whereas activated mast cells were more abundant in the high-risk group (Figure 8C). Suggesting that mast cell status may significantly affect LUSC prognosis. Furthermore, by analysing immune cell correlations in LUSC patients, we gained a deeper understanding of the tumor microenvironment (TME) (Figure 8D), The correlation between model genes and immune cells also provided new insights into potential immunotherapeutic strategies for LUSC (Figure 8E). Lastly, we assessed the immune score, stromal,and estimated scores in high-and low-risk groups based on profiling, revealing significant differences in the tumour microenvironment (TME) between the two groups (Figure 8F).

### 4.6 Prognostic analysis of LUSC patients

**FIGURE 9:**
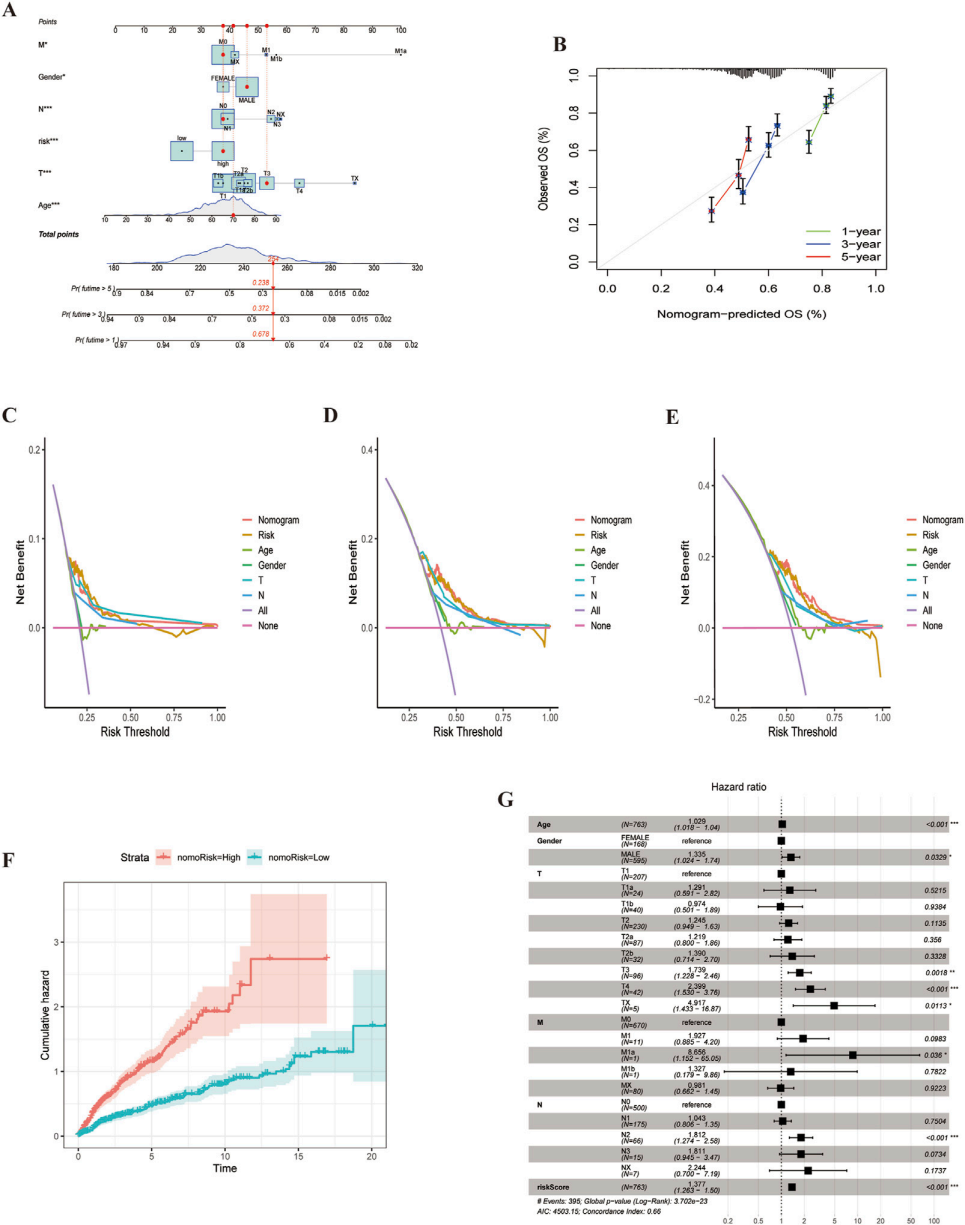
Nomogram for LUSC patients. **(A)** Nomogram constructed based on ARGs scores and clinicopathologic features.**(B)** Calibrated Nomogram. **(C–E)** DCA evaluation of LUSC patients prognosis.**(F)** Risk curves showing survival probability progression of over time.**(G)** Forest plot of multivariate Cox regression analysis, Illustrating the association between clinical characteristics and risk scores for LUAD patients.

We developed a nomogram to predict 1-, 3-, and 5-year survival, incorporating clinicopathological features and ARGs risk scores (Figure 9A). The calibration plots demonstrated high predictive accuracy (Figure 9B). DCA results indicated that the nomogram was a strong predictor of survival outcomes in LUSC patients (Figures 9C–E). Additionally, cumulative risk curves showed that patients survival risk increased over time, regardless of whether they were in the high or low-risk group (Figure 9F). Forest plot analysis revealed that risk score, age and stage were the primary factors influencing LUSC prognosis (Figure 9G). These findings underscore the robustness of the risk score-based nomogram for predicting survival in LUSC patients.

### 4.7 Model gene survival analysis and drug sensitivity

**FIGURE 10:**
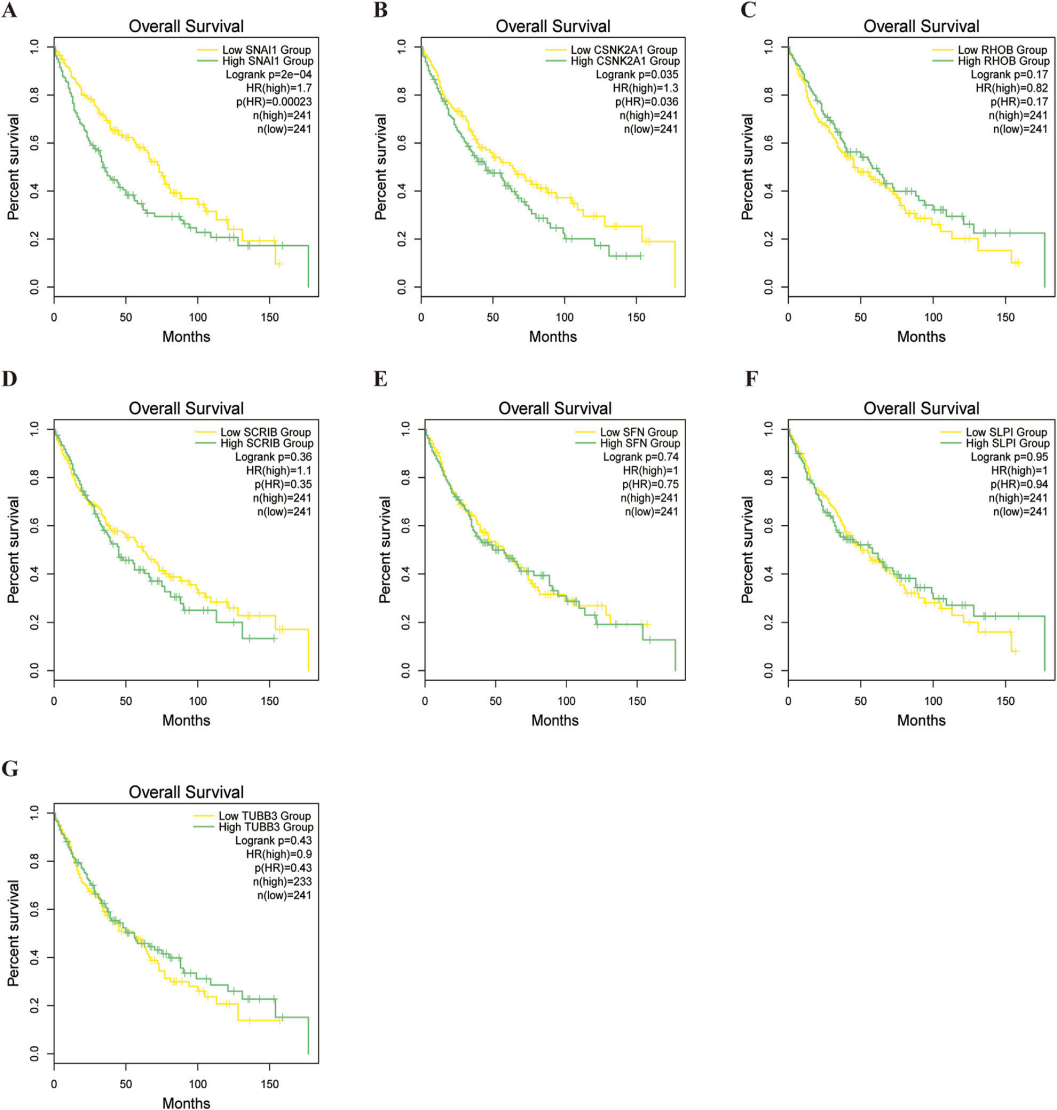
Survival analysis of model genes.

To evaluate differences in drug sensitivity among LUSC patient subgroups,we conducted a drug sensitivity analysis, we analysed tumour sensitivity to drugs. The results indicated that most drugs was less effective in the high-risk group, although some exhibited increased sensitivity (Supplementary Table S8; Supplementary Figures S2–S12). Subsequently, Survival analysis of the model genes in LUSC patients revealed that CSNK2A1 (P = 0.035) and SNAI1 (P = 0.0002) significant impacted prognosis. High expression of these genes was strongly associated with elevated mortality in LUSC patients (Figures 10A–G).

### 4.8 Virtual screening and molecular dynamics simulation

**FIGURE 11:**
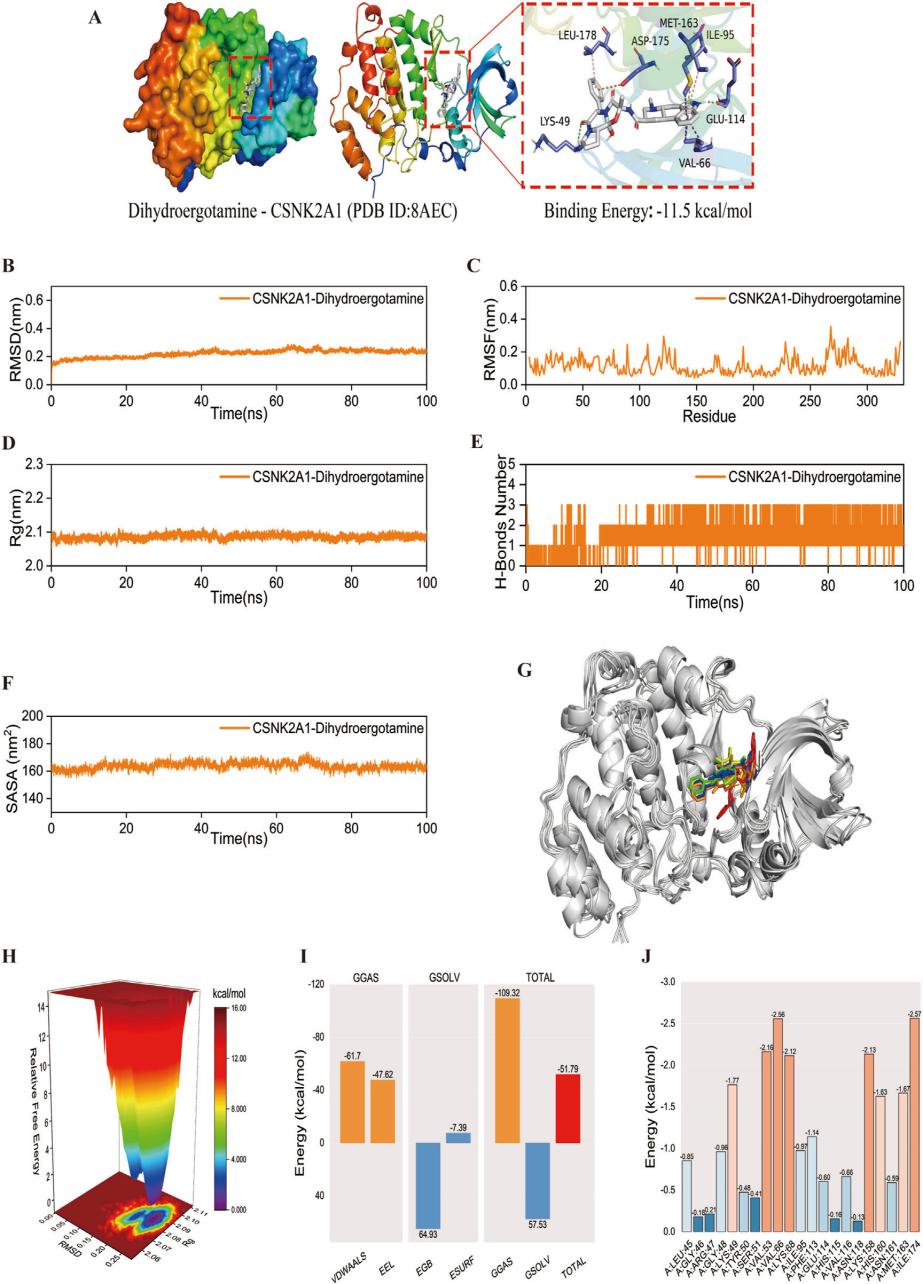
Molecular docking and molecular dynamics simulation. **(A)** The docking result of the CSNK2A1-Dihydroergotamine complex. **(B–F)** The curve of CSNK2A1 protein and Dihydroergotamine complex: RMSD, RMSF, Rg, Hydrogen bond analysis, and SASA. Curves for the CSNK2A1 protein and the Dihydroergotamine complex. **(G)** Comparison of conformation of the complex at five different molecular dynamics simulation time points. **(H)** Free energy distribution. **(I)** Average binding free energy. **(J)** Contributions of amino acid residues involved in binding.

Although SNAI1 and CSNK2A1 are both pivotal, SNAI1 is unsuitable for virtual screening due to it’s small peptide nature. Conversely, CSNK2A1, being an enzyme protein, is an ideal target for virtual screening and molecular docking. We chose CSNK2A1 as the receptor protein for virtual screening, and the results indicated that Dihydroergotamine exhibited the strongest affinity with CSNK2A1. Molecular docking analysis revealed a binding energy of 11.5 kcal/mol between CSNK2A1 and Dihydroergotamine, signifying a very strong binding affinity. The docking visualisation, generated using PyMOL 2.3.0 (Figure 11A), demonstrated that Dihydroergotamine was tightly bound to multiple amino acid residues within CSNK2A1 via various interactions, with binding energies below −7.2 kcal/mol, suggesting a significant impact on CSNK2A1 structure, function, and biological activity. Molecular dynamics analysis confirmed the stability of the CSNK2A1-Dihydroergotamine complex, with RMSD, RMSF, Rg, and hydrogen bonding analyses indicating that minimal fluctuation for the complex (Figures 11B–H). Binding energy analysis revealed strong free energy (−51.79 kcal/mol) (Figure 11I). Finally, binding energy contribution analysis showed that ILE-174 and VAL-66 were critical in the binding of Dihydroergotamine to CSNK2A1, aligning with molecular docking results and further verifying the complex’s high stability (Figure 11J).

### 4.9 Effect of Dihydroergotamine on LUSC cells

**FIGURE 12:**
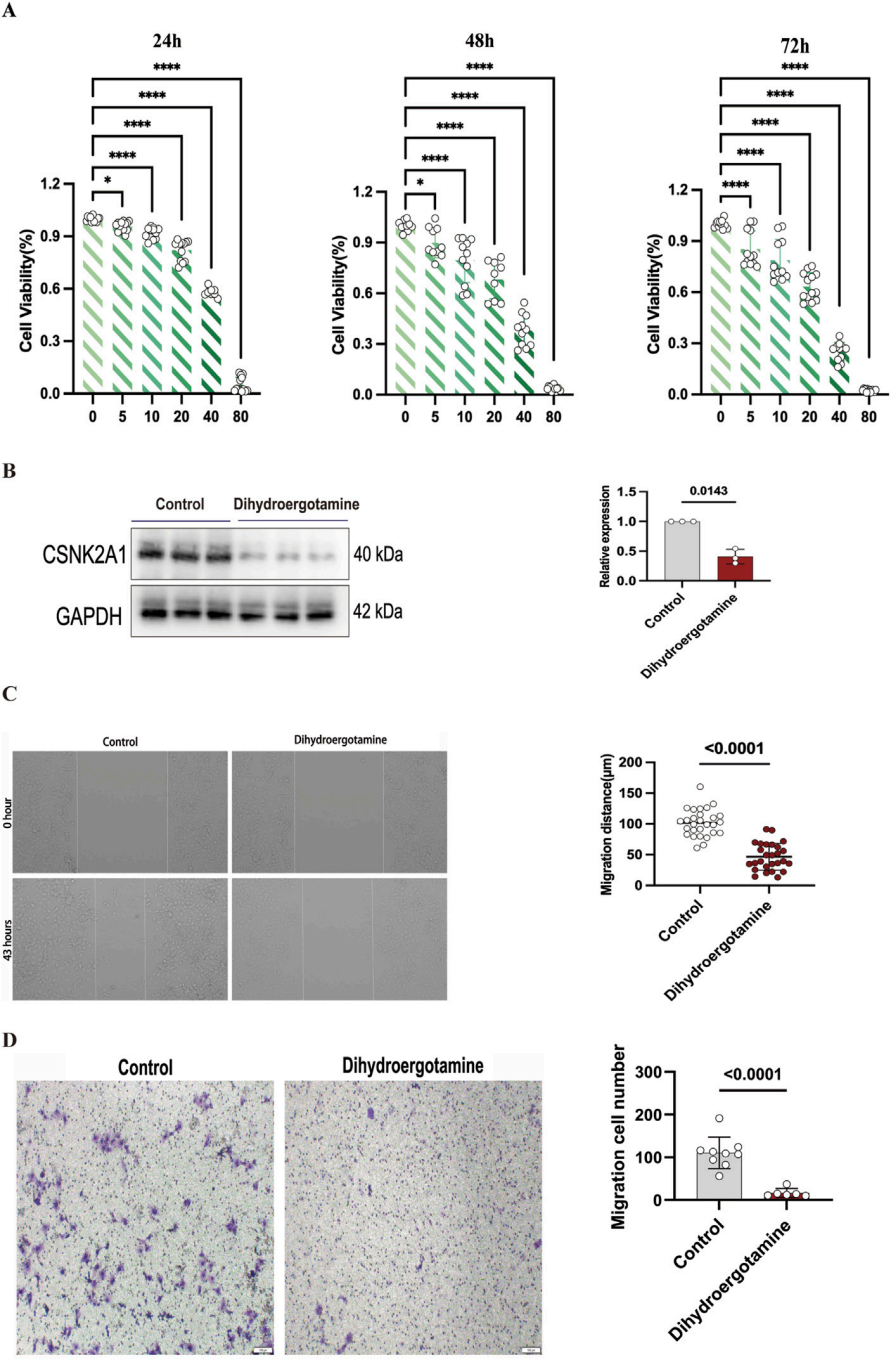
Effect of Dihydroergotamine on NCI-H2170 cells viability, CSNK2A1 expression, migration, invasion. **(A)** Inhibition of NCI-H2170 cells viability by Dihydroergotamine. **(B)** Changes in CSNK2A1 expression in NCI-H2170 cells after Dihydroergotamine treatment. **(C)** Effect of 40 μM Dihydroergotamine on the migration of NCI-H2170 cells. **(D)** Effect of 40 μM Dihydroergotamine on the invasion of NCI-H2170 cells.

The effect of Dihydroergotamine on the viability of LUSC cells was evaluated using the CCK-8 assay, and its dose-dependent anti-proliferative activity was investigated. The results indicated that Dihydroergotamine exhibited a significant inhibitory effect on LUSC cell viability (Figure 12A). Specifically, Dihydroergotamine demonstrated pronounced inhibitory activity within the short-term incubation period (24 h). However, as the incubation time increased (48–72 h), the inhibitory effect in the low-concentration groups gradually diminished. Based on these findings, the optimal inhibitory concentration of 40 μM was selected for subsequent experiments.

### 4.10 Western blot analysis of CSNK2A1 expression

Western blot analysis revealed that the expression level of CSNK2A1 was significantly reduced following treatment with Dihydroergotamine (Figure 12B). These results suggest that CSNK2A1 has potential as a prognostic biomarker for LUSC, and Dihydroergotamine shows promise as a potential therapeutic agent for LUSC.

### 4.11 Migration and invasion assays

Wound healing and transwell assays were conducted to evaluate the effects of Dihydroergotamine on the migration and invasion abilities of LUSC cells. Compared to the control group, treatment with 40 μM Dihydroergotamine significantly reduced the wound healing rate of NCI-H2170 cells after 43 h (P < 0.01) (Figure 12C). Similarly, the transwell assay results demonstrated that the number of NCI-H2170 cells penetrating through the Matrigel-coated membrane was significantly decreased following treatment with 40 μM Dihydroergotamine (P < 0.01) (Figure 12D).

## 5 Discussion

LUSC is one of the common subtypes of NSCLC and presents a major public health challenge due to its treatment difficulties and poor prognosis, leading to extremely high mortality rates (Lau et al., 2022),Although several prognostic markers for LUSC have been identified (Šutić et al., 2024; Wang et al., 2024), Pprognostic models associated with anoikis have not been deeply explored.

Recent studies have highlighted the critical role of abnormal cell death in tumor initiation and progression. For instance, Xie et al. (2024) identified the disulfidptosis regulator, glycogen synthase 1 (GYS1), as an effective therapeutic target in triple-negative breast cancer. Similarly, ferroptosis-related mRNAs and lncRNAs have been identified as ideal prognostic biomarkers in gastric cancer (Liu Y. et al., 2023). In this study, we developed an anoikis-related prognostic model for LUSC to provide new insights into its diagnosis and treatment. Unlike previous studies based on a single dataset, we integrated data from the TCGA and GSE30219 datasets to enhance the model’s accuracy and reliability. Using this model, we identified eight anoikis-related genes (ARGs) closely associated with LUSC and confirmed the significance of CSNK2A1 and SNAI1 in LUSC prognosis through survival analyses. Therefore, this study constructed a prognostic model for LUSC related to anoikis, offering new insights into the diagnosis and treatment of LUSC. Unlike previous studies that relay on a single database, we integrated the TCGA and GSE30219 datasets to improve the model’s accuracy and reliability. Based on this model, we identified eight ARGs closely linked to LUSC prognosis and confirmed through survival analysis the significance of CSNK2A1 and SNAI1 in LUSC patients survival.

Casein kinase 2α1 (CSNK2A1) encodes the protein kinase CK2α, which phosphorylates various proteins and regulates biological processes such as the cell cycle and apoptosis, also affecting the Wnt/β-catenin signalling pathway, which plays an important role in cancer (Chua et al., 2017; Gao and Wang, 2006). Studies have shown that knockdown CSNK2A1 expression in KRAS-mutant lung cancer cells inhibits cancer cell proliferation and Wnt/β-catenin signalling (Wang et al., 2019); Yu et al. used machine learning algorithms to screen genes related to mitochondrial autophagy in NSCLC and established a prediction model that includes CSNK2A1 (Yu et al., 2023). While the role of CSNK2A1 in lung cancer has been established, its specific function in LUSC has yet to be fully investigated.

Snail family transcriptional inhibitory protein 1 (SNAI1) is a zinc-finger transcription factor that downregulates E-calmodulin expression through specific recognition of its promoter and is closely associated with tumour development (Singh et al., 2021). Although studies on SNAI1 in lung cancer are limited, it has been shown that upregulation of miR-34a-5p and subsequent downregulation of SNAI1 induce apoptosis in lung cancer cells (Aida et al., 2021). Further analysis revealed that LUSC patients had higher survival rates when SNAI1 expression was low, suggesting that SNAI1 may be a key gene in LUSC (Chawhan and Dsouza, 2024). Despite limited research, SNAI1’s critical role in other tumours has been confirmed, highlighting the need for further exploration.

Studies have shown that CSNK2A1 and SNAI1 correlate significantly with immune cells, particularly M0 macrophages and activated mast cells. M0 macrophages are considered resting macrophages that differentiate into M1 and M2 subtypes (Zhang et al., 2022). Recent studies suggest that macrophages in gliomas maintain a continuum between M1 and M2 phenotypes, which are associated with M0 macrophages (Gabrusiewicz et al., 2016). M0 macrophages have been significantly linked to poor prognosis in high-grade gliomas (Huang et al., 2020). Although the relationship with LUSC is unclear,their tumourigenic role in gliomas suggests that further study is warranted, Mast cells, traditionally associated with allergic and inflammatory responses (Bischoff, 2007). Have recently been shown to play a key role in shaping the tumour microenvironment (Aponte-López and Muñoz-Cruz, 2020). Found in the microenvironment of solid tumours, they influence cancers such as oesophageal and ovarian cancers (Wang et al., 2013; Chan et al., 2005); while playing a negative role in lung adenocarcinoma and breast cancer (Takanami et al., 2000; Reddy et al., 2019). Mast cells release tumour necrosis factor-alpha (TNF-α) and IL-1, which directly affect tumour pathogenesis (Déry et al., 2000; Litmanovich et al., 2018). Given the crucial role of M0 macrophages and mast cells in the tumour microenvironment, the associations between CSNK2A1,SNAI1,and these immune cells deserve further investigation.

In small molecule drug screen based on CSNK2A1, Dihydroergotamine demonstrated strong binding affinity to CSNK2A1, and molecular dynamics simulations indicated good stability of the CSNK2A1-Dihydroergotamine complex, This suggests could serve as a potential therapeutic option for LUSC. Dihydroergotamine is an by the FDA-approved ergot alkaloid derivative primarily used for the treating migraine (Bigal and Tepper, 2003; Hernández-Rodríguez et al., 2024). Given challenges of development new anticancer drugs, repurposing existing drugs for cancer treatment holds significant value. Recent studies have found that Dihydroergotamine can target colon cancer via JAK2 (Chandrasekhar et al., 2024). Therefore, it’s potential therapeutic role in LUSC warrants further exploration.

Despite the development of a predictive model for LUSC prognosis based on a risk score nomogram, several limitations remain. First, although this is the first predictive model for LUSC related to anoikis-associated genes, the model lacks validation through novel methodologies. Second, while the interaction between Dihydroergotamine and CSNK2A1 has been confirmed, its clinical efficacy in LUSC requires further *in vitro* and *in vivo* validation. Third, although we conducted a limited number of cellular experiments to assess the impact of Dihydroergotamine on LUSC cells and observed a significant reduction in CSNK2A1 expression, there is a lack of clinical sample-based immunohistochemical studies. Fourth, the absence of *in vivo* experiments to evaluate the drug’s tumor-suppressive effects further limits the findings.

## 6 Conclusion

Overall, our study is the first to establish a risk model for LUSC treatment and prognosis based on anoikis-related genes. Further analysis indicates that these genes offer reliable predictive accuracy in LUSC and have a significant impact on its immune microenvironment, demonstrating promising immunological characteristics. Notably, virtual screening and molecular dynamics studies suggest the potential of CSNK2A1 as a future therapeutic target for LUSC, while Dihydroergotamine shows promise as a potential drug for LUSC treatment. Cellular experiments also validated the findings derived from bioinformatic analysis. In conclusion, CSNK2A1 may serve as a reliable prognostic indicator for the survival of LUSC patients, and its immunological characteristics position it as a potential target for immunotherapy. Moreover, the application of Dihydroergotamine in LUSC warrants further exploration.

## Data Availability

The datasets presented in this study can be found in online repositories. The names of the repository/repositories and accession number(s) can be found in the article/ Supplementary Material.
