# Supplementary material for: Identification of potential diagnostic targets and therapeutic strategies for anoikis-related biomarkers in lung squamous cell carcinoma using machine learning and computational virtual screening
Source: Front Pharmacol. 2025 Feb 14;16:1500968. doi: 10.3389/fphar.2025.1500968 (PMC11868076; doi:10.3389/fphar.2025.1500968)
Supplement: Supplementary file 2 [file DataSheet2.docx]

**A novel predictive model for anoikis-related gene markers in squamous cell carcinoma of the lung based on machine learning and single-cell analysis, revealing their immune relevance.**

**Supplementary figures**

**
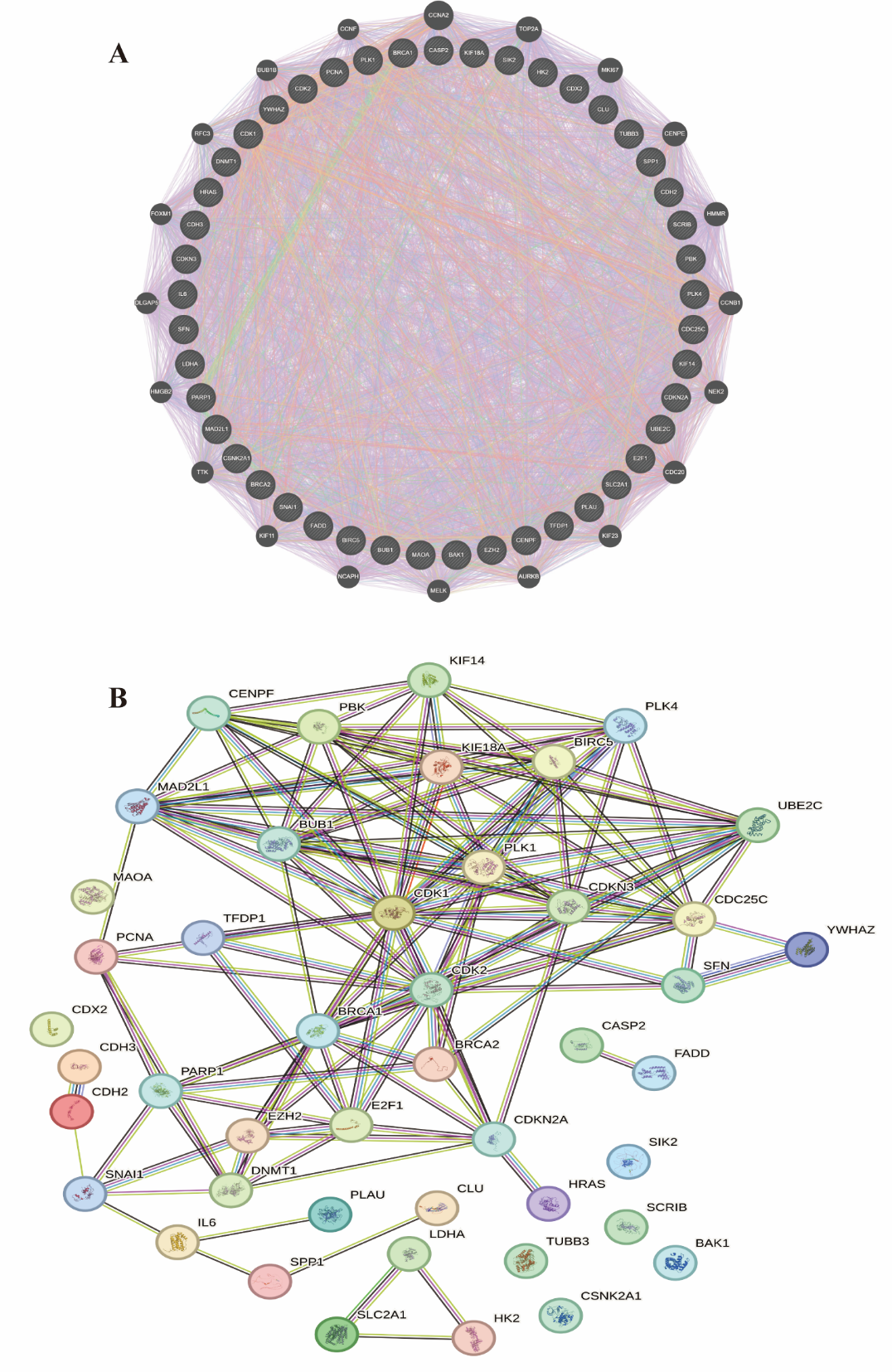
**

**Fig S1**

network diagram. (A) Network diagram of 45 prognostic genes interacting with anoikis closely related genes. (B) Protein Interaction PPI Network Diagram.


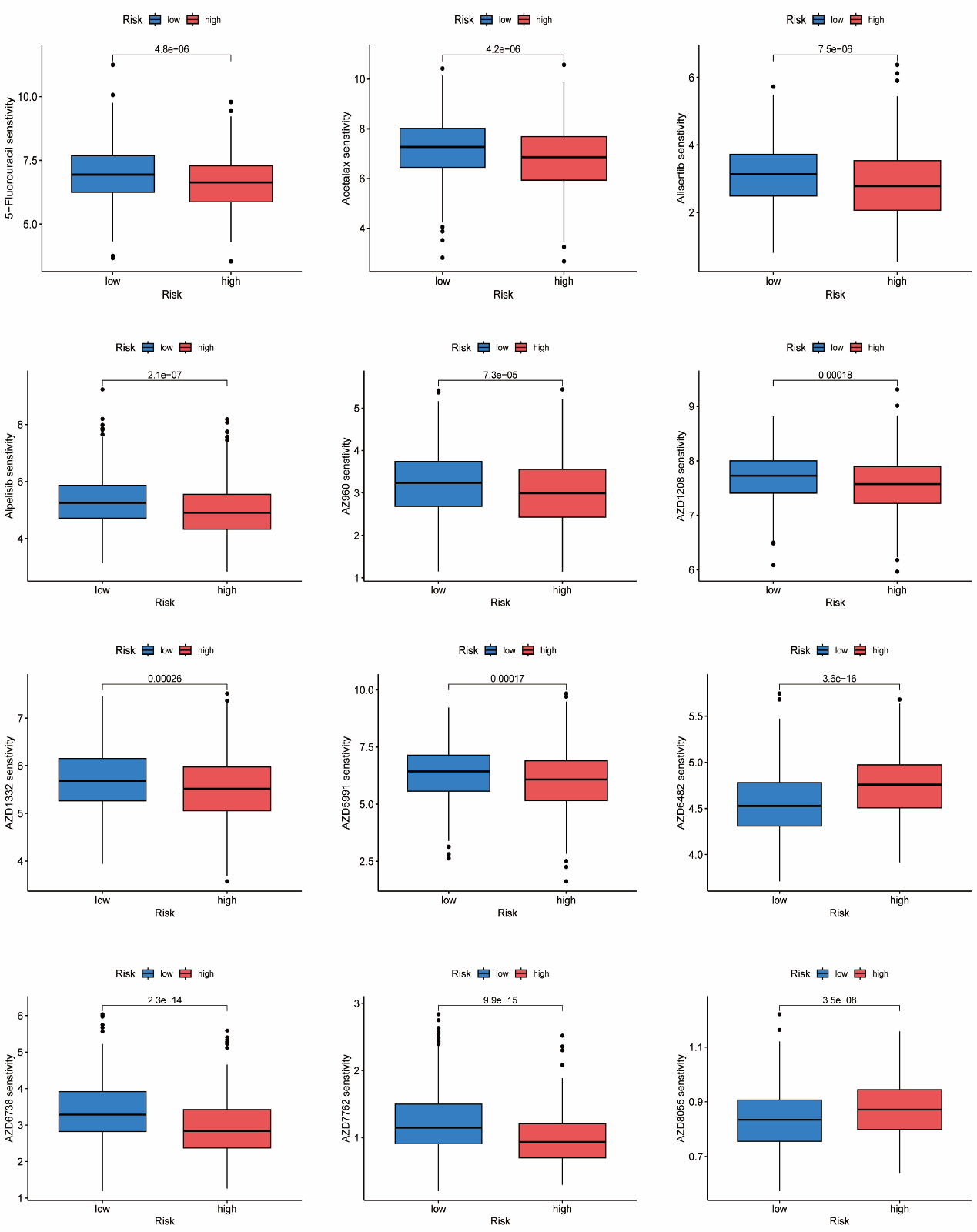


**Fig. S2**

LUSC drug sensitivity.


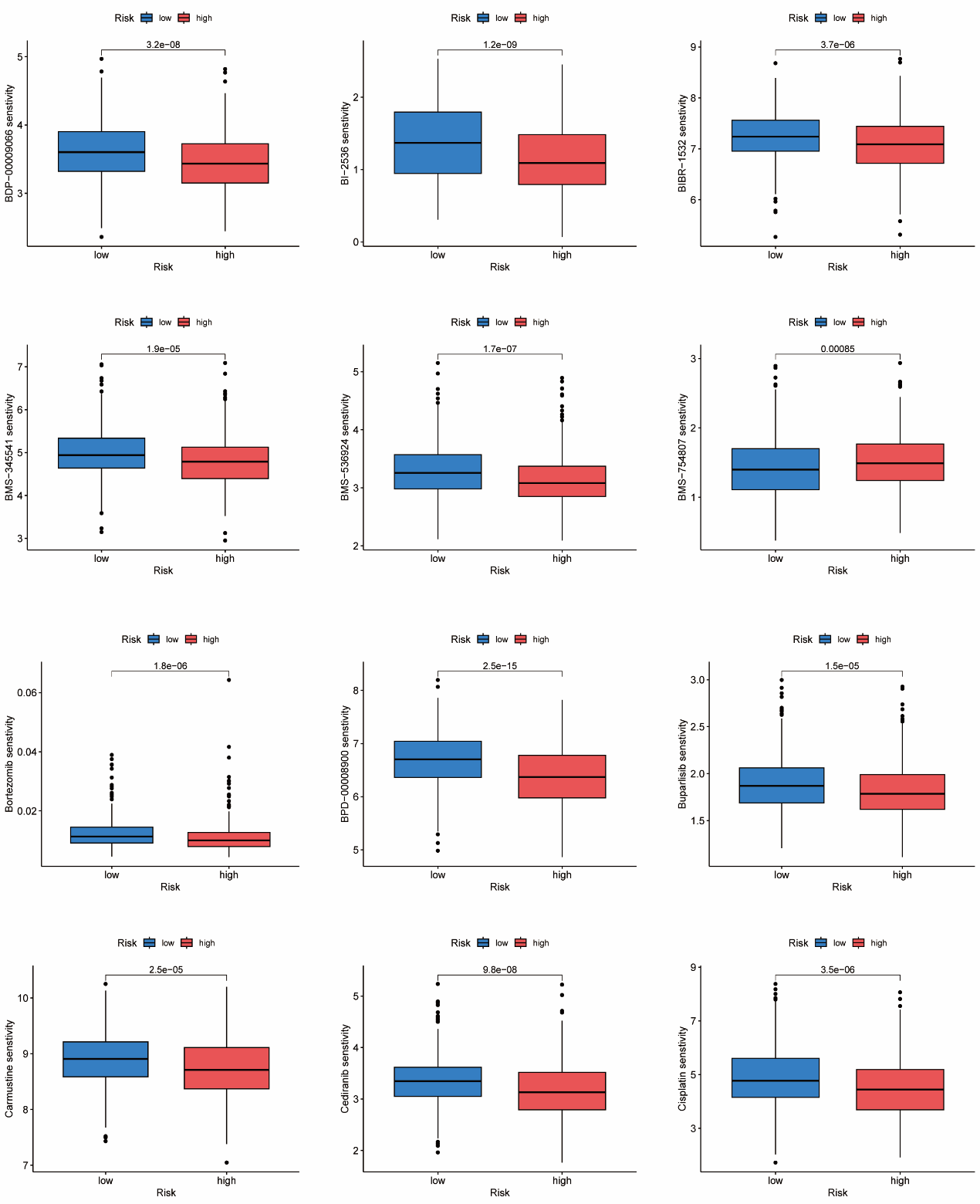


**Fig. S3**

LUSC drug sensitivity


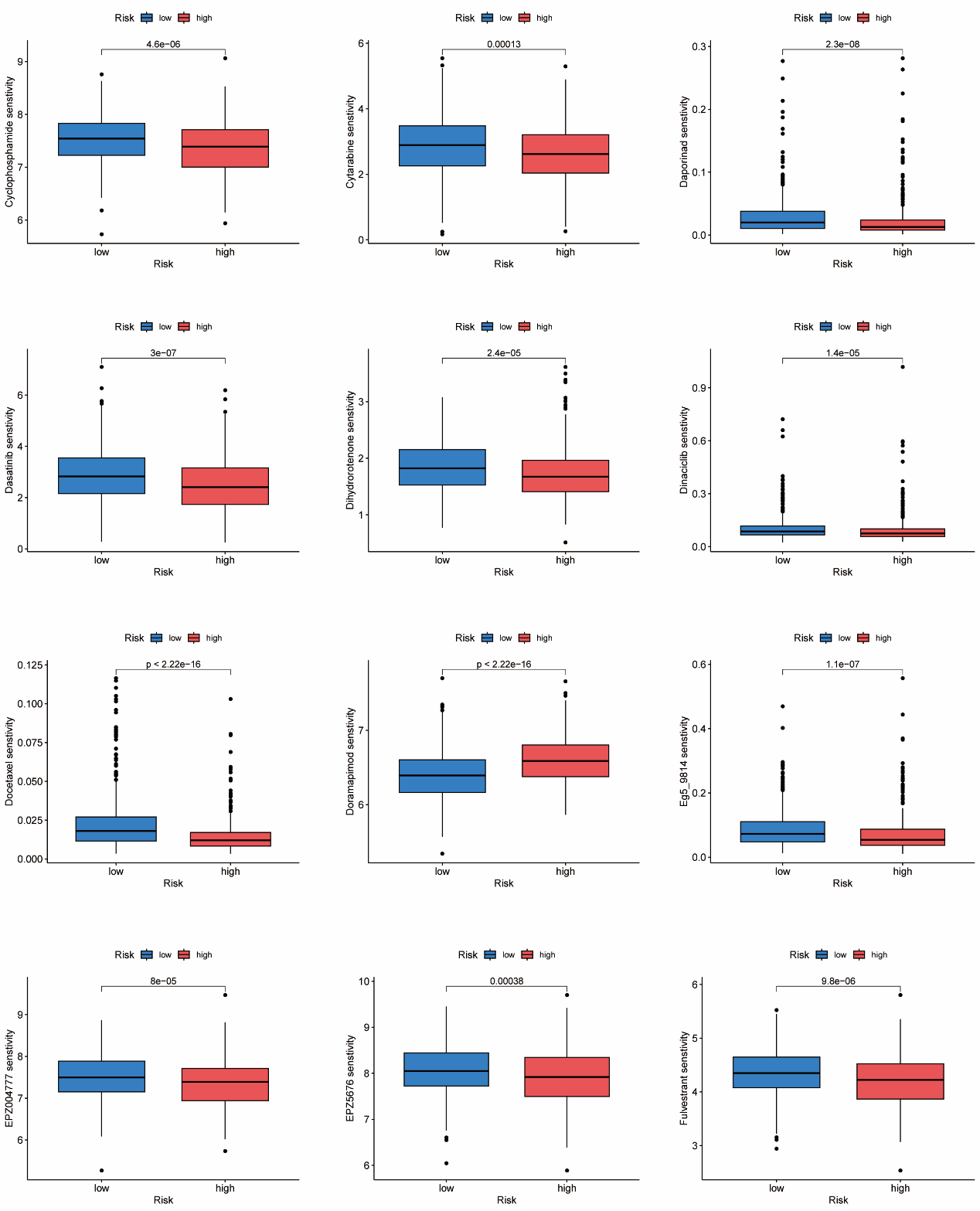


**Fig. S4**

LUSC drug sensitivity.


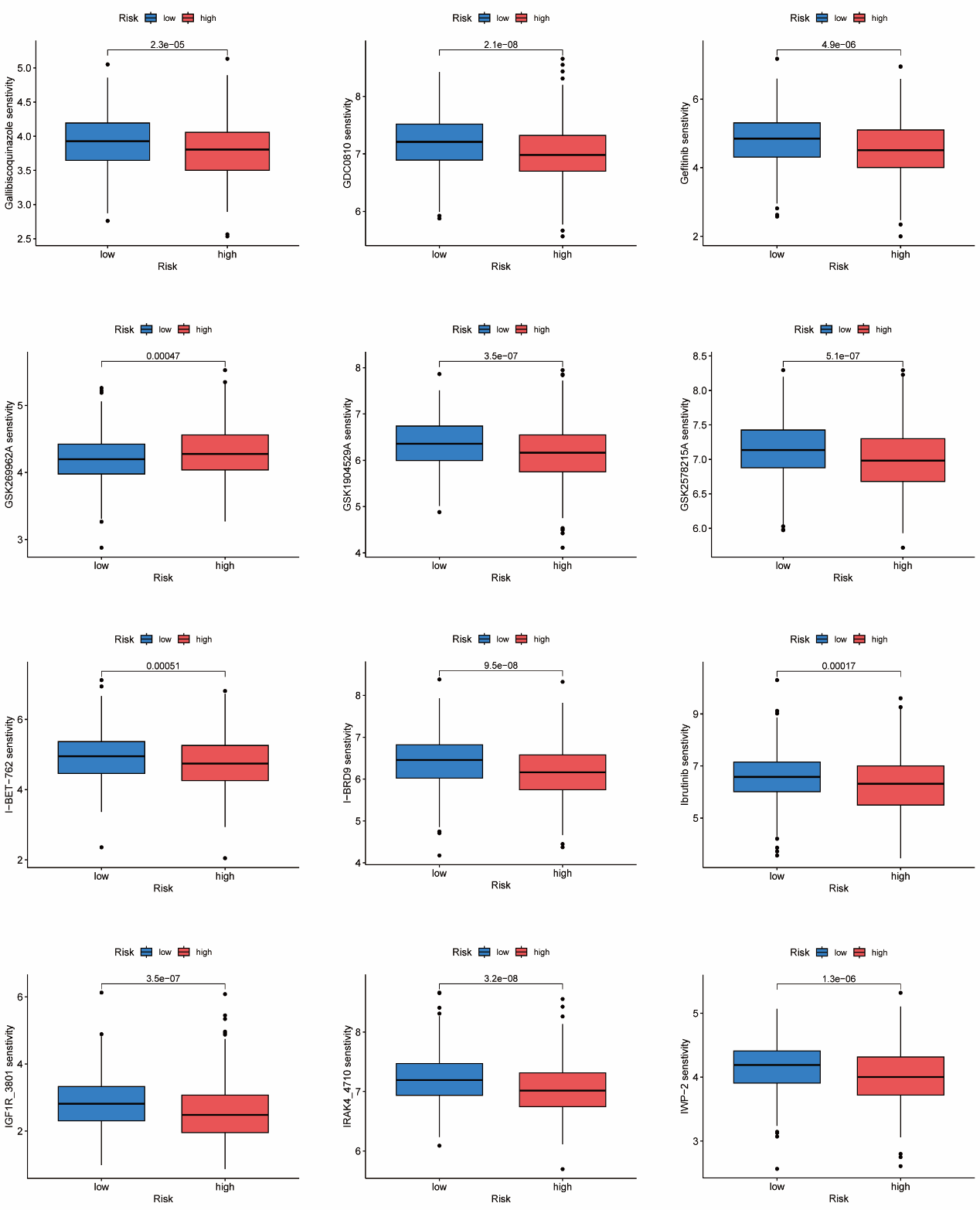


**Fig. S5**

LUSC drug sensitivity.


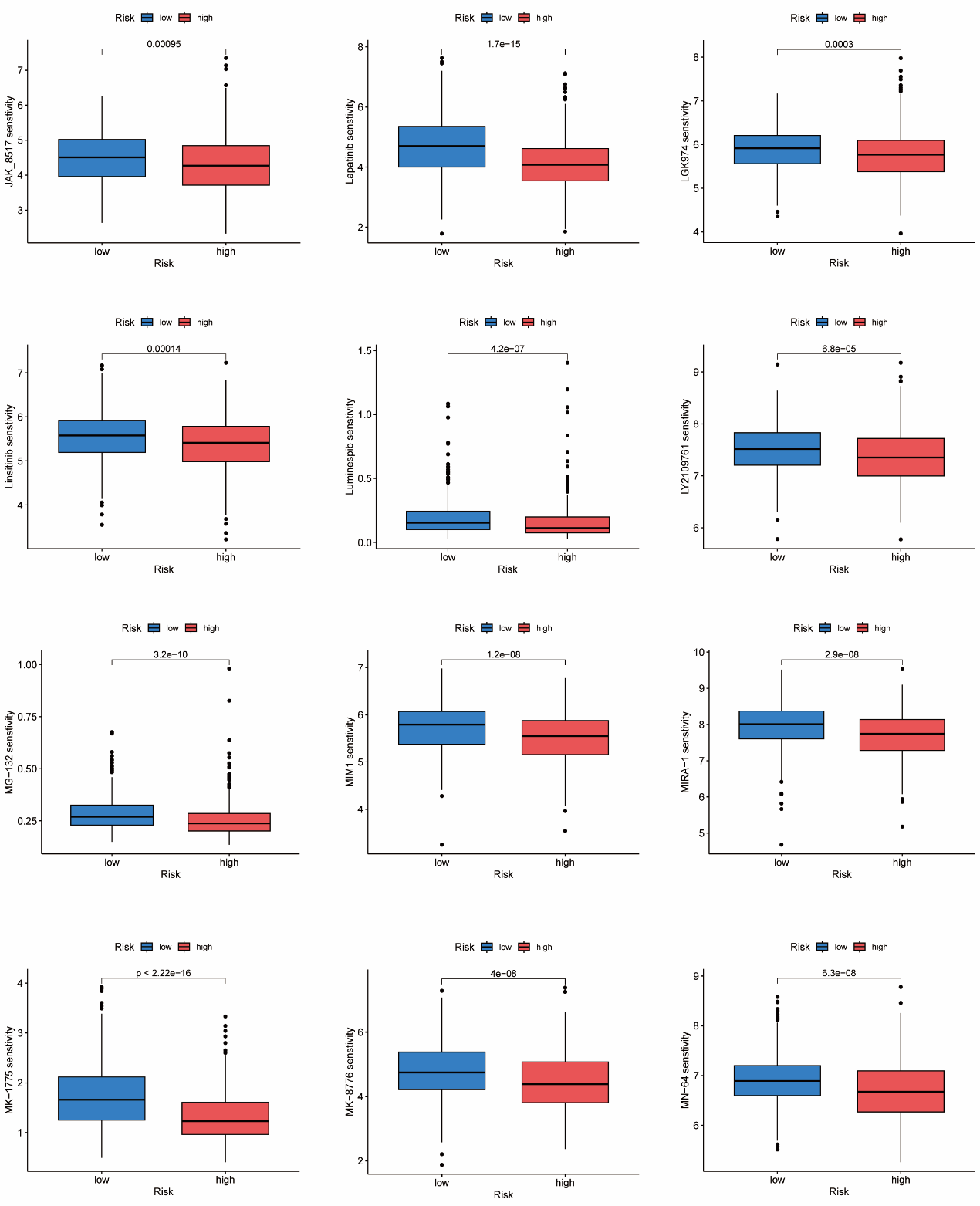


**Fig. S6**

LUSC drug sensitivity.


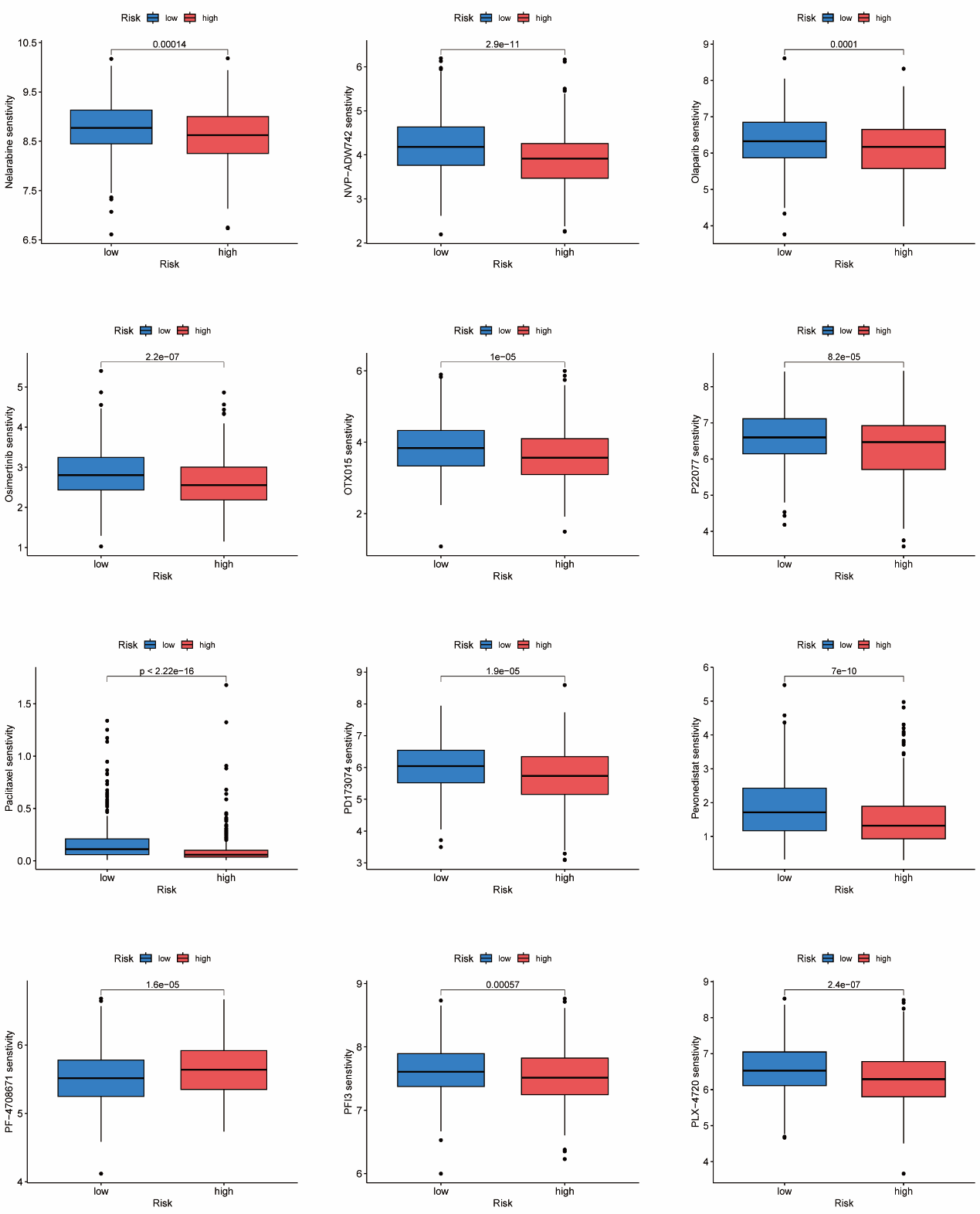


**Fig. S7**

LUSC drug sensitivity.


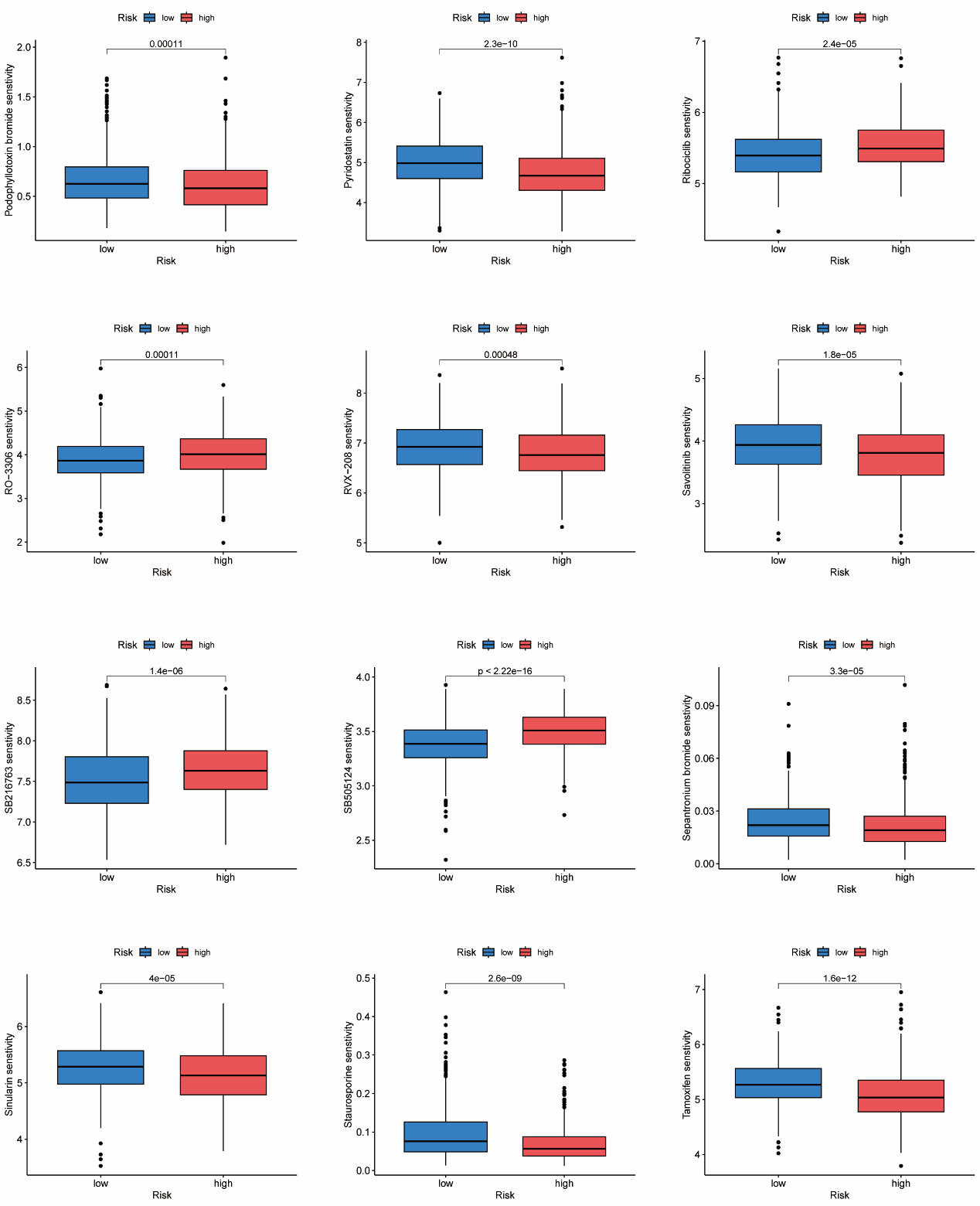


**Fig. S8**

LUSC drug sensitivity.


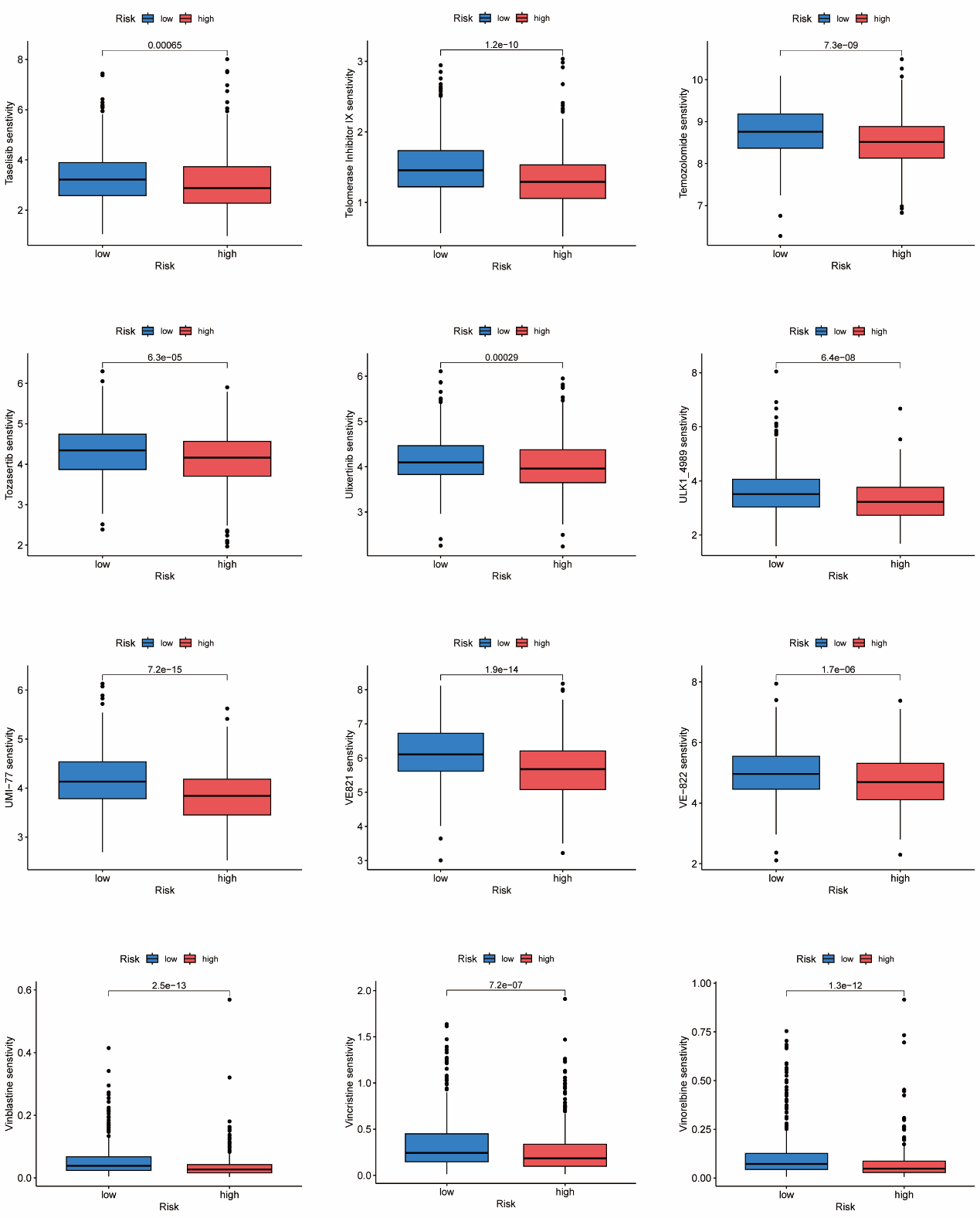


**Fig. S9**

LUSC drug sensitivity.


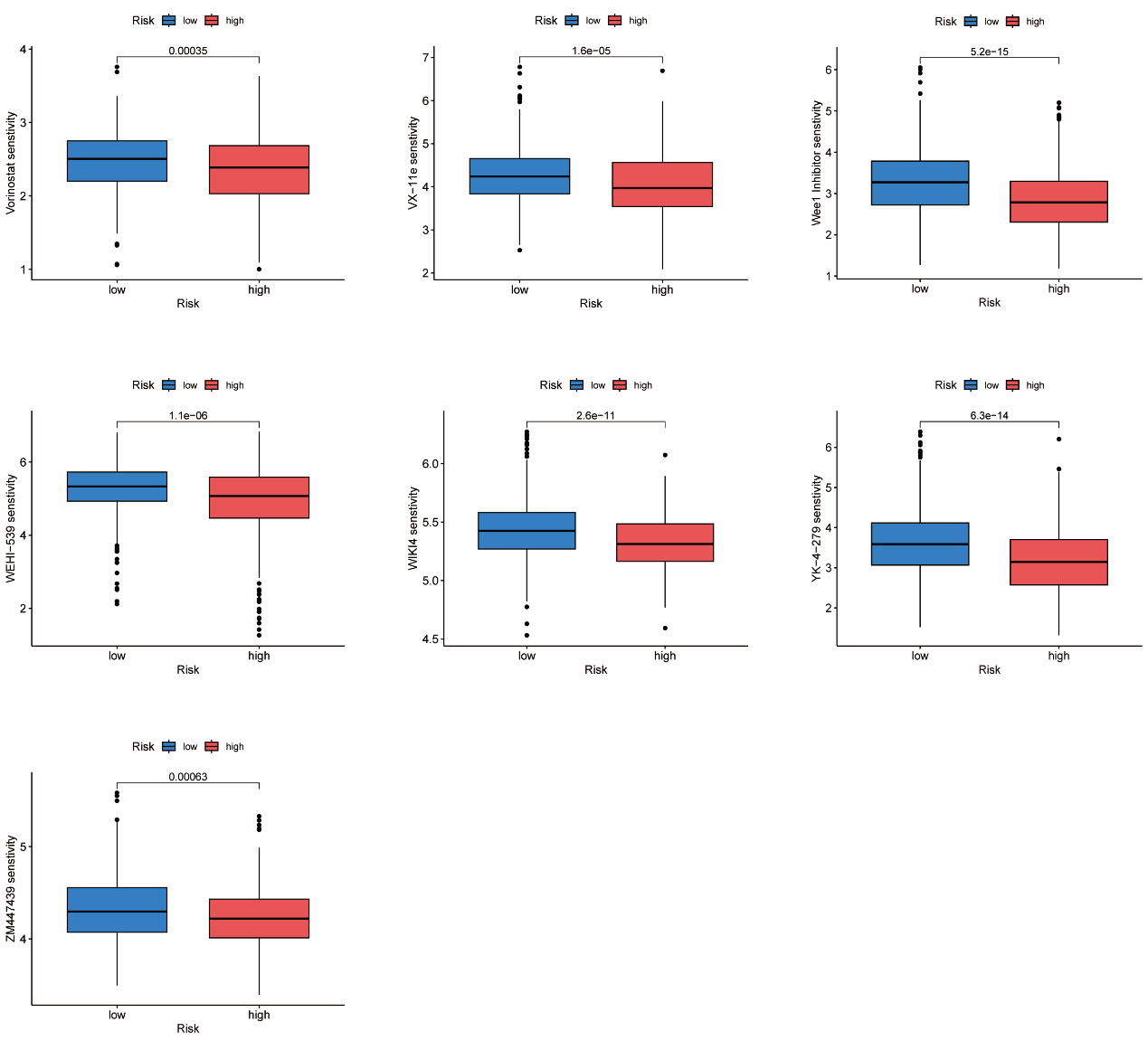


**Fig. S10**

LUSC drug sensitivity.
